# Virus Biomimetic-Delivery Systems for the Production of Vaccines

**DOI:** 10.3390/biomimetics11020150

**Published:** 2026-02-18

**Authors:** Marcela-Elisabeta Barbinta-Patrascu, Irina Negut, Bogdan Bita

**Affiliations:** 1 Department of Electricity, Solid-State Physics and Biophysics, Faculty of Physics, University of Bucharest, 405 Atomistilor, 077125 Magurele, Romania; marcela.barbinta@unibuc.ro; 2 National Institute for Laser, Plasma and Radiation Physics, 409 Atomistilor, 077125 Magurele, Romania; 3 Optospintronics Department, National Institute of Research and Development for Optoelectronics-INOE 2000, 409 Atomistilor, 077125 Magurele, Romania

**Keywords:** virus-like particles (VLPs), virosomes, vaccine development, biomimetic nanoparticles, recombinant expression systems, nanotechnology in vaccines, scalable vaccine platforms

## Abstract

The persistent emergence of infectious diseases has underscored the critical demand for next-generation vaccine technologies that are safe, effective, and scalable. This review explores virus biomimetic delivery systems, focusing on virus-like particles (VLPs) and virosomes as promising platforms for vaccine and therapeutic development. VLPs are self-assembled nanostructures composed of viral structural proteins that mimic native virions without carrying genetic material, while virosomes are reconstituted viral envelopes that retain functional glycoproteins but lack a nucleocapsid. Both systems provide strong immunogenicity and safety by mimicking viral architecture while eliminating the risk of replication. The paper examines various expression platforms for VLP production, including bacterial, yeast, insect, mammalian, and plant-based systems, highlighting their respective advantages, challenges, and optimization strategies. Mechanistic insights into antigen presentation, immune activation, and cellular uptake pathways are discussed to explain their superior performance in eliciting humoral and cellular immune responses. Furthermore, current applications of VLPs and virosomes in vaccines against major pathogens such as SARS-CoV-2, influenza, Newcastle disease virus, malaria, hepatitis, and respiratory syncytial virus are reviewed, demonstrating their versatility and clinical potential. By integrating molecular engineering, nanotechnology, and biofabrication strategies, virus biomimetic systems represent a transformative frontier in vaccinology, immunotherapy, and targeted drug delivery.

## 1. Introduction

The continuous emergence of infectious diseases has highlighted the urgent need for innovative vaccine technologies that offer enhanced safety, efficacy, and scalability. Virus-like particles (VLPs) and virosomes have emerged as leading biomimetic platforms for vaccine development, drug delivery, and immunotherapy [1]. These systems mimic the structural and immunogenic properties of viruses while eliminating the risks associated with viral replication, providing a potent and versatile approach to preventive and therapeutic interventions. VLPs are self-assembling nanostructures composed of viral proteins, whereas virosomes are reconstituted viral envelopes that retain functional glycoproteins, offering unique advantages in antigen presentation and immune activation [1,2].

Recent advances in molecular engineering, expression systems, and nanotechnology have expanded the applications of VLPs [2] and virosomes across a wide spectrum of diseases, including COVID-19 [3], HPV [4], and emerging zoonotic threats [5].

Numerous reviews have contributed to understanding these platforms. Recent works have detailed VLP design and immunological properties [4,6], and allergen-specific immunotherapy approaches [7]. Other reviews have examined VLP immunogenic mechanisms and viral vaccine applications [8], VLP vaccine formulation and production optimization [9], and advances in scalable expression systems and assembly control [10,11,12].

The literature on virosomes has also expanded, focusing on lipid-based viral mimetics, antigen encapsulation, and mucosal immunization [13,14,15]. Meanwhile, recent developments in hybrid nanoparticle and RNA-based vaccine systems [16,17,18] highlight the growing convergence between biomimetic and genetic vaccine technologies.

Despite this rich body of work, most existing reviews analyze VLPs and virosomes independently, without providing an integrated comparative perspective. The present manuscript uniquely offers a side-by-side analytical synthesis of both platforms, examining their structural features, expression systems, immunogenic properties, and translational performance. Furthermore, we critically evaluate their disease-specific applications (e.g., COVID-19, influenza, RSV, malaria, hepatitis, and NDV) and identify practical criteria for selecting between these systems based on antigen characteristics, scalability, and target immune response. This comparative framework aims to bridge the gap between mechanistic understanding and applied vaccine design.

## 2. VLPs

VLPs are nanoscale self-assembling structures composed of viral structural proteins that resemble authentic viruses in morphology but lack genetic material. As a result, VLPs are non-infectious and non-replicative, making them ideal candidates for use in vaccines, drug delivery, and diagnostic applications [19].

VLPs are formed when viral capsid or envelope proteins spontaneously self-assemble into virus-like nanostructures. These particles maintain the antigenic characteristics of their parent viruses, allowing them to elicit strong immune responses without the risk of infection [10]. Depending on the virus from which they are derived, VLPs can be classified as enveloped or non-enveloped. Enveloped VLPs include components of the lipid membrane derived from the host cell, while non-enveloped VLPs are solely made up of capsid proteins.

### 2.1. Classification of VLPs

**Figure 1:**
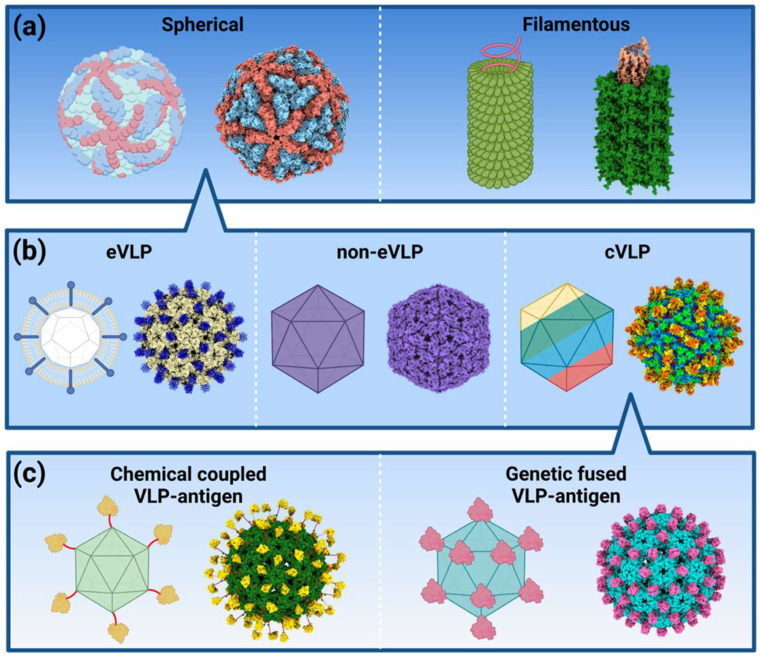
Schematic and structural representation of VLP classification: (**a**) VLPs are categorized by morphology as either spherical or filamentous. (**b**) Within spherical shape, structural distinctions are made between enveloped VLPs (eVLPs; membrane shown in white, glycoproteins in blue), non-enveloped VLPs (non-eVLPs; capsid in purple), and chimeric VLPs (cVLPs; capsid in rainbow). (**c**) cVLPs are further subclassified based on conjugation method, either chemical coupling (capsid in green, linker in red, epitope in yellow) or genetic fusion (capsid in light blue, epitope in pink Created in Biorender. Marcela Barbinta (2025) BioRender.com and ChimeraX v1.17 [7].

VLPs are categorized based on their structural composition and presence of a lipid envelope (Figure 1):

Enveloped VLPs: These VLPs incorporate a lipid bilayer derived from host cells, which includes embedded viral glycoproteins. These glycoproteins mimic the surface properties of native viruses, allowing enveloped VLPs to closely resemble infectious virions in their immunogenic characteristics. Examples of enveloped VLPs include HIV and influenza VLPs, both of which have been extensively studied for vaccine development. Enveloped VLPs present additional challenges in production due to the complexity of assembling membrane-bound proteins and maintaining structural stability [20].

Non-enveloped VLPs: They lack a lipid bilayer and are composed solely of viral capsid proteins which self-assemble into highly ordered, virus-like structures. These VLPs are typically more stable and easier to manufacture than enveloped VLPs (which incorporate host-derived lipid membranes), making them ideal for large-scale vaccine production. Hepatitis B and human papillomavirus (HPV) VLPs are in this category, and both have been successfully implemented in licensed vaccines such as Engerix-B^®^ and Gardasil^®^. Non-enveloped VLPs have demonstrated high immunogenicity without the need for an adjuvant, which has contributed to their widespread use in commercial vaccines [21].

Chimeric VLPs are nanoscale constructs that integrate structural proteins from different viral serotypes, enabling modifications to the VLP core with antigens and the encapsulation of various therapeutic or diagnostic agents. These VLPs offer several advantages, including the ability to present foreign epitopes, deliver diagnostic molecules, and transport multiple therapeutic agents while facilitating targeted delivery to specific cells, tissues, or organs. A notable study demonstrated the synthesis of chimeric VLPs incorporating the M1 capsid proteins from the influenza A/swine flu/Iowa/15/30/H1N1 virus, produced within silkworms [22]. These VLPs were further engineered to feature a glycosylphosphatidylinositol-anchored single-chain variable fragment, allowing them to selectively target colon carcinoma cells. Additionally, they were utilized as carriers for doxorubicin delivery at a concentration of 13.7 nM. Despite their potential, the production of chimeric VLPs is influenced by several factors, including glycosylation patterns, steric hindrance, protein conjugation efficiency, antigen size, and the choice of host cell for expression [22].

Recent advances in VLP research have explored modifications to improve their immunogenicity and stability. For enveloped VLPs, techniques such as lipid composition engineering and glycoprotein stabilization have enhanced their structural integrity and expression efficiency. Meanwhile, non-enveloped VLPs have benefited from protein engineering approaches that enhance self-assembly and antigenic presentation, leading to improvements in vaccine efficacy [10].

### 2.2. Production and Engineering of Virus-like Particles

VLPs are produced through recombinant expression systems that allow viral structural proteins to self-assemble into virus-like structures while remaining non-infectious due to the absence of genetic material. These particles can be generated in various biological systems, including bacteria, yeast, insect cells, mammalian cells, and plant-based expression platforms. The selection of an expression system depends on the complexity of the VLP structure, the need for post-translational modifications, and scalability requirements [23].

#### 2.2.1. Bacterial Expression Systems

Bacterial expression systems, such as *E. coli*, are frequently used for experimental production of non-enveloped VLPs due to their simplicity and cost-effectiveness [24]. These systems enable straightforward cloning, transformation, and expression of viral structural proteins, making them suitable for early-stage vaccine research and proof-of-concept studies. However, *E. coli* lacks the post-translational modification machinery required for proper folding and glycosylation of complex viral proteins, which can limit the structural authenticity and immunogenicity of VLPs. Consequently, for large-scale production and clinical-grade applications, alternative expression platforms such as yeast (*Saccharomyces cerevisiae*, Gardasil^®^) and insect cells (baculovirus expression vector system, Cervarix^®^) are preferred. These eukaryotic systems provide superior protein processing, correct disulfide bond formation, and high-fidelity VLP assembly, enabling efficient manufacturing of safe and immunogenic vaccines [25,26,27,28].

One of the key advantages of bacterial expression systems is the ease of genetic manipulation. Bacterial platforms provide a convenient system for modifying genetic constructs, facilitating the engineering of chimeric and fusion VLPs for vaccine development. Advances in genetic engineering have enabled the incorporation of multiple viral antigens into a single VLP, improving immunogenicity and broadening vaccine applicability [29]. Furthermore, bacterial cultures grow rapidly and can be easily scaled up to meet industrial production demands, making them a preferred choice for large-scale VLP manufacturing [21].

Despite these advantages, bacterial expression systems present several challenges and limitations. One major drawback is the lack of post-translational modifications, such as glycosylation, which are crucial for the proper function of many viral proteins, particularly those from enveloped viruses. The absence of glycosylation can impact the stability and immunogenicity of VLPs, necessitating alternative expression systems for certain applications [30]. Additionally, some viral capsid proteins have a tendency to form insoluble inclusion bodies, which require complex refolding procedures to restore functionality. This can lead to reduced yields and increased production costs [31].

Another challenge associated with bacterial expression systems is endotoxin contamination. Since *E. coli* produces endotoxins, extensive purification steps are required to remove these potentially harmful byproducts and ensure the safety of VLP-based vaccines and therapeutics. Optimized purification strategies, such as affinity chromatography and endotoxin removal columns, are often necessary to achieve high-purity VLPs suitable for human use [32].

To overcome these challenges, several optimization strategies have been explored. One approach involves the co-expression of molecular chaperones to enhance the proper folding of viral capsid proteins, thereby improving VLP solubility and yield. Studies have demonstrated that co-expression strategies can significantly reduce the formation of inclusion bodies and improve the efficiency of bacterial-based VLP production [33]. Additionally, alternative bacterial hosts such as *Bacillus subtilis* have been explored as safer expression platforms, given their lower endotoxin levels and ability to support self-assembled VLP production. Research has indicated that *B. subtilis* offers an improved safety profile while maintaining high expression levels [30].

#### 2.2.2. Yeast Systems

Yeast expression systems, including *Pichia pastoris* and *Saccharomyces cerevisiae*, have been widely utilized for the production of VLPs due to their ability to support proper protein folding, glycosylation, and scalable fermentation. These systems provide a eukaryotic expression platform that ensures the correct post-translational modifications of viral structural proteins, making them highly suitable for vaccine and therapeutic applications [34]. Unlike bacterial systems, yeast cells can perform glycosylation and properly fold complex proteins, which is critical for the stability and immunogenicity of certain VLPs [35]. One of the most successful applications of yeast-based VLP production is the commercial manufacture of Hepatitis B virus (HBV) VLPs, as seen in the Engerix-B^®^ and Recombivax HB^®^ vaccines.

Yeast-based recombinant expression systems are highly scalable and cost-effective, making them an attractive option for industrial vaccine production [36]. In addition to being cost-efficient, yeast cultures present a low risk of contamination with human pathogens, offering enhanced biosafety compared to mammalian cell systems [37]. These advantages have positioned yeast as a reliable system for large-scale VLP manufacturing.

Recent advancements in yeast-based VLP engineering have focused on improving immunogenicity and expanding vaccine applications. One promising approach involves chimeric VLPs for multivalent vaccines, which use the L-A virus capsid of yeast (*Saccharomyces cerevisiae*) for the assembly of hybrid VLPs capable of encapsulating multiple antigens. This strategy has been shown to enhance immune responses and broaden vaccine applicability [37]. Additionally, yeast systems have been successfully used for the high-yield production of Polyomavirus VLPs, demonstrating their effectiveness in gene therapy and vaccine applications [38].

Despite their advantages, yeast-based expression systems present certain challenges. One of the main issues is hyperglycosylation, where yeast-derived glycosylation patterns differ from those in human cells, potentially affecting antigenicity. To overcome this, advances in glycoengineering are being developed to modify yeast glycosylation pathways to resemble those of mammalian cells [39]. Another challenge is optimization of VLP secretion and yield, as some yeast-expressed VLPs remain intracellular, requiring additional steps for extraction and purification. Research is ongoing to enhance secretory VLP production and simplify purification strategies [39].

#### 2.2.3. Insect Cell Systems

Insect cell expression systems, particularly the Baculovirus Expression Vector System (BEVS), have become a well-established platform for the production of VLPs used in vaccine development and gene therapy. These systems are capable of efficiently producing influenza, HPV, and Japanese encephalitis virus (JEV) VLPs, among others. The insect cell lines most commonly used for BEVS are *Spodoptera frugiperda* (Sf9) and *Trichoplusia ni* (High Five™), which enable the large-scale production of recombinant proteins with proper folding and post-translational modifications [20].

BEVS enables the large-scale production of VLPs with high expression yields, making it an efficient and scalable system for vaccine manufacturing. The adoption of single-use (SU) bioreactors has further improved the scalability of VLP production using insect cells [40]. Unlike bacterial systems, BEVS provides authentic post-translational modifications, including glycosylation, which is essential for the proper antigenic properties of VLPs. The ability to co-express multiple proteins simultaneously makes it particularly useful for producing complex VLPs, such as those for HIV, influenza, and Ebola vaccines [41].

BEVS has been successfully applied in human and veterinary vaccines, including Cervarix^®^ (HPV) and Flublok^®^ (influenza), and has also been utilized for the production of gene therapy vectors, such as Adeno-Associated Virus vectors [42]. The A baculoviral TB (Transcriptional Booster) expression cassette has been shown to increase VLP yields by 300%, making BEVS-based vaccine production more cost-effective [40]. Furthermore, bioprocess optimization for influenza vaccines has led to the development of the VLP-factory™ system, which allows high-throughput production of influenza VLPs and incorporates functional mutations to enhance immune responses [41].

In addition to its vaccine applications, BEVS is now being utilized to produce Adeno-associated virus (AAV)-like particles for human gene therapy, enabling efficient vector production [43]. Despite its advantages, BEVS faces several challenges, including differences in glycosylation patterns between insect cells and mammalian cells, which may affect antigenicity. Glycoengineering approaches are being developed to humanize glycosylation pathways and enhance the immunogenicity of insect cell-produced VLPs [44]. Although insect cell systems are highly scalable, improvements in bioprocess automation and the integration of single-use bioreactors are needed to further enhance commercial production [45].

The BEVS remains a powerful tool for VLP-based vaccine production and gene therapy vector development. With successful commercial applications, including Cervarix^®^ and Flublok^®^, BEVS continues to be one of the most promising platforms for large-scale biopharmaceutical manufacturing. Future advancements in bioprocess optimization and glycoengineering will further enhance its role in next-generation vaccine production and therapeutic applications.

#### 2.2.4. Mammalian Cell Systems

Mammalian cell expression systems provide the most authentic post-translational modifications, making them the preferred platform for the production of enveloped VLPs, including those derived from HIV, Ebola, and coronaviruses. Unlike bacterial or insect cell systems, mammalian cells such as Human Embryonic Kidney (HEK293), Chinese Hamster Ovary (CHO), and Vero cells are capable of producing VLPs with native-like glycosylation patterns, ensuring proper antigen presentation and enhanced immunogenicity [46].

Mammalian cells have been successfully used to produce VLPs for influenza, with studies showing that influenza A and B VLPs produced in mammalian cell platforms generate robust immune responses and induce functional antibodies [47]. Additionally, quadrivalent hepatitis C VLPs have been produced using a mammalian system, demonstrating the feasibility of large-scale production for clinical applications [48].

Despite these advantages, mammalian cell-based VLP production is associated with high production costs and lower yields compared to other expression platforms. This is due to the slower growth rate of mammalian cells, complex culture conditions, and the need for expensive bioreactors. However, recent advancements in process intensification have significantly improved VLP yields. For example, the development of an inducible HEK-293 stable cell line enabled a 60-fold increase in volumetric yield of influenza VLPs [49]. Similarly, the use of modified vaccinia virus Ankara (MVA) vectors has led to the production of highly immunogenic mammalian-derived influenza VLPs, which closely resemble live viruses in antigen presentation [50].

Mammalian VLP production is also being explored for next-generation polio vaccines, providing an alternative to traditional live-attenuated vaccines. Recent research has demonstrated that thermostabilized polio VLPs produced in mammalian cells can elicit neutralizing antibodies similar to inactivated poliovirus vaccines, offering a promising approach for post-eradication polio immunization [51].

#### 2.2.5. Plant-Based Systems

Plant-based expression systems offer an innovative and scalable approach to the production of VLPs for vaccine development. These systems utilize plants such as *Nicotiana benthamiana* and algae-based platforms like *Chlamydomonas reinhardtii* to produce recombinant proteins in a cost-effective and safe manner. Unlike mammalian and bacterial expression systems, plant-based systems eliminate the risk of contamination from human pathogens, making them a promising alternative for large-scale VLP production [52].

The transient expression of VLPs in plants is a rapidly evolving technology that allows for fast, high-yield production. The use of agroinfiltration, in which *Agrobacterium*-mediated gene delivery introduces viral structural proteins into plant cells, has enabled the rapid production of vaccine candidates. This system has been successfully applied to produce influenza, hepatitis B, and norovirus VLPs [53]. For example, tobacco-based transient expression platforms have been explored for the production of Hepatitis B core antigen (HBcAg) VLPs in green algae *Chlamydomonas reinhardtii*, demonstrating their potential for vaccine development [52].

Plant-based systems require minimal infrastructure compared to mammalian cell culture, significantly reducing production costs. They can be rapidly scaled up for mass production, making them ideal for pandemic preparedness [54]. The DNA replicon system enables high-yield VLP production in just a few days, as demonstrated for Norwalk virus and hepatitis B VLPs [55]. The absence of human or animal-derived contaminants enhances biosafety and simplifies regulatory approval [52].

Recent advances in plant-based VLP engineering have demonstrated significant progress in vaccine development. Research has shown that fluorescent-tagged influenza VLPs produced in *N. benthamiana* can be used to study immune response mechanisms [56]. Additionally, the plant-based expression of Bluetongue virus VLPs has provided effective protection in livestock, demonstrating the potential of plant-made vaccines for veterinary applications [57].

Despite its advantages, plant-based VLP production faces challenges such as differences in glycosylation patterns, which may affect antigenicity. However, glycoengineering strategies are being developed to humanize plant-derived glycoproteins, improving their compatibility with human immune responses [52]. Ongoing clinical trials of plant-produced influenza vaccines suggest that plant-based VLPs will play a growing role in next-generation vaccine development [53].

Plant-based expression systems represent a cost-effective, scalable, and safe platform for VLP production. With advancements in transient expression technology and glycoengineering, plant-based vaccines are increasingly viable for human and veterinary applications. Continued research and development in this field will further expand the role of plant-made VLPs in global vaccine production and pandemic preparedness.

### 2.3. Mechanism of Action of VLPs

VLPs immunogenic properties make them highly effective vaccine platforms, capable of eliciting strong humoral and cellular immune responses [58]. VLPs act as potent immunogens due to their repetitive surface geometry, which enhances antigen presentation and immune activation [59].

Upon administration, VLPs are rapidly recognized by antigen-presenting cells (APCs) such as dendritic cells (DCs) and macrophages. The highly ordered and repetitive structure of VLPs facilitates their uptake via pattern recognition receptors (PRRs), triggering innate immune responses [60,61]. Once internalized, VLPs are processed and their antigens are displayed on major histocompatibility complex (MHC) molecules, leading to the activation of T-helper cells (CD4^+^) and cytotoxic T-cells (CD8^+^) [61].

VLPs effectively induce both humoral and cell-mediated immunity. After uptake by antigen-presenting cells, VLP-derived antigens are processed and presented on MHC-II molecules, leading to B-cell activation and antibody production that are crucial for neutralizing viral infections. In parallel, some VLP antigens can enter the cross-presentation pathway and be displayed on MHC-I molecules, thereby activating CD8^+^ cytotoxic T lymphocytes (CTLs). This coordinated activation of B and T cells promotes long-lasting immune memory, making VLPs highly suitable for both prophylactic and therapeutic vaccine development [61].

One of the key advantages of VLPs is their ability to traffic into lymph nodes efficiently, which enhances their interaction with immune cells. Studies using optical imaging techniques have demonstrated that VLPs accumulate in lymphoid tissues, where they promote robust germinal center reactions and antibody affinity maturation [61,62]. Furthermore, adjuvant formulations can be used to enhance VLP immunogenicity, improving vaccine efficacy against challenging pathogens such as HPV, hepatitis B, and SARS-CoV-2 [61,62].

VLP-based vaccines have demonstrated high immunogenicity and safety, leading to their approval for human papillomavirus (HPV), hepatitis B (HBV), and malaria (Mosquirix™) vaccines [61,62]. Their structural flexibility allows the incorporation of heterologous antigens, making them promising platforms for chimeric vaccines targeting multiple pathogens or diseases, including cancer immunotherapy [61,62].

## 3. What Are Virosomes?

**Figure 2:**
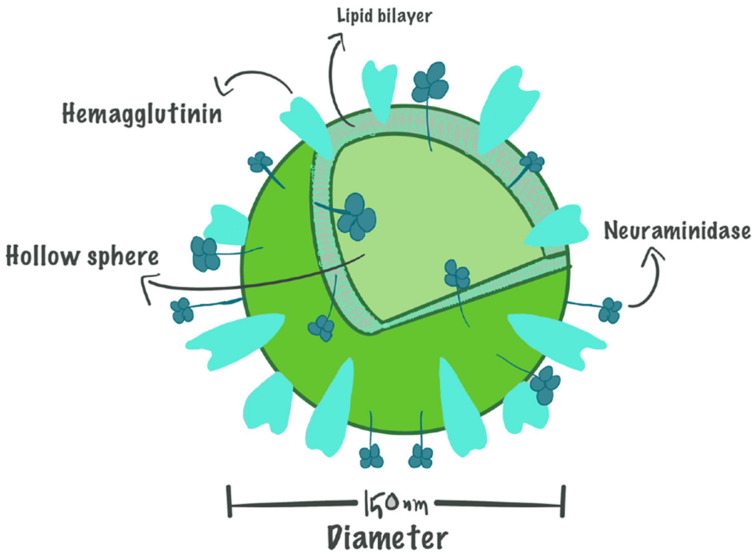
The structure of a virosome [64].

Virosomes are spherical, unilamellar vesicles (60–200 nm) composed of viral envelope phospholipids with the nucleocapsid removed, effectively eliminating the risk of viral replication while retaining functional properties that enhance targeted drug and antigen delivery [63]. At the same time, virosomes are biomimetic, biodegradable, and biocompatible bio-entities that show a high safety profile in vaccine development [63]. The structure of a virosome can be seen in Figure 2 [64].

A significant advantage of virosomes is their ability to adsorb epitopes of antigens through hydrophobic interactions or lipid linkers, both on their surface and within their phospholipid bilayer, allowing for efficient antigen presentation [63].

Virosomes mimic natural viral structures by incorporating viral glycoproteins such as hemagglutinin (HA) and neuraminidase (NA) into their membrane, providing an effective system for antigen delivery. These glycoproteins can be displayed on the virosome surface or located inside the hollow membrane vesicles, thereby preserving the ability of the virosome to interact with immune system components [64].

Hydrophilic drugs can be encapsulated within the hollow interior of virosomes, while hydrophobic agents integrate into the phospholipid bilayer, increasing their bioavailability and improving targeted delivery [64]. Additionally, surface modifications using hydrophilic polymers such as polyethylene glycol (PEG) and polyvinylpyrrolidone (PVP) have been shown to extend circulation time, reducing rapid clearance by the immune system [65].

Moreover, virosomes offer the advantage of active targeting by incorporating specific ligands, antibodies, or peptides that enable precise interactions with tumor cells, respiratory epithelial cells, and immune system components. This targeting capability is particularly relevant in vaccine development and immunotherapy, as it facilitates the efficient uptake of antigens by APCs, leading to robust immune activation [66].

While virosomes provide notable advantages in terms of biocompatibility, targeted delivery, and flexibility for incorporating multiple antigens or adjuvants, VLPs have achieved broader clinical translation and commercial success. Several licensed vaccines, including Gardasil^®^ (HPV) and Engerix-B^®^ (HBV), demonstrate the proven efficacy, scalability, and regulatory maturity of VLP-based platforms in large-scale immunization programs [64]. In contrast, VLPs have a rigid protein-based framework that can limit antigen movement, particularly when multiple antigenic sites are closely packed [67]. By anchoring protein antigens to the fluidic phospholipid bilayer of virosomes, interactions with host cell receptors are enhanced, improving immune system recognition and response [64].

Virosomes can be engineered with diverse antigenic epitopes, allowing them to target different types of host cells. They are typically internalized by host cells at a neutral pH, but their fusion with the endosomal membrane occurs in a pH-dependent manner, requiring acidic conditions to trigger the process [13].

### 3.1. Difference Between Virosomes and VLPs

Both virosomes and VLPs are engineered nanostructures used in vaccine development and drug delivery. However, they differ in their structure, composition, and mechanism of action (Table 1).

Both systems are highly effective in vaccine development, with VLPs being more widely used due to their structural stability and ease of production, while virosomes are valuable for targeted drug delivery and fusion-based vaccines.

### 3.2. Preparation of Virosomes

The production of virosomes involves a series of precise steps to ensure proper assembly while maintaining the biological activity of the viral glycoproteins. These steps include virus solubilization, nucleocapsid removal, reconstitution into lipid bilayers, and surface functionalization.

#### 3.2.1. Virus Solubilization

The first step in virosome production is the solubilization of the viral envelope, which is crucial for separating viral lipids and proteins while maintaining functional glycoproteins [13]. The solubilization of viral envelopes is a critical step in the production of virosomes, ensuring that functional viral glycoproteins are preserved while disrupting the viral membrane. Various detergents are employed in this process, each with specific effects on viral components.

Several studies have explored the use of different detergents to solubilize viral glycoproteins. Octyl-beta-D-glucopyranoside (OG) has been widely used for influenza virus glycoprotein extraction, with studies showing that it can effectively solubilize HA while preserving its antigenic properties for over two years [76]. Similarly, MESK, a novel nonionic detergent, has demonstrated selective solubilization of glycoproteins from enveloped viruses such as influenza, parainfluenza, and herpes viruses, retaining their biological activity and immunogenicity [77].

Other examples are Triton X-100, octyl glucoside, and 1,2-dicaproyl-sn-glycero-3-phosphocholine (DCPC), which effectively disrupt the viral membrane and allow the extraction of essential viral components [78]. The choice of detergent significantly affects the stability and functionality of viral glycoproteins. A study on rabies virus glycoprotein (RVG) solubilization found that Triton X-100 was less effective than CHAPS and octyl s-(+)-glucopyranoside (OGP) in extracting functional glycoproteins [79]. In contrast, nonionic detergents such as Cymal-5 have shown instability at room temperature but improved stability at lower temperatures or in the presence of entry inhibitors [80]. Short-chain phospholipids like 1,2-dihexanoylphosphatidylcholine (DHPC) have been used; DCPC dissolves viral membranes and permits subsequent removal and reconstitution of membrane components into virosomes with morphological and fusogenic properties comparable to native envelopes [81].

The efficiency of solubilization depends on detergent concentration, time of exposure, and temperature, all of which must be optimized to prevent denaturation of viral proteins.

#### 3.2.2. Nucleocapsid Removal

The removal of the nucleocapsid is a crucial step in virosome production, ensuring that the resulting particles are non-infectious and suitable for vaccine and drug delivery applications [64]. This process involves ultracentrifugation, which efficiently separates the viral core from solubilized membrane components.

Ultracentrifugation has been widely used for nucleocapsid removal, and its efficiency depends on the precise selection of speed, duration, and gradient composition. Studies have optimized the ultracentrifugation conditions for different viruses. For instance, research on Newcastle disease virus (NDV) found that the highest nucleocapsid protein yield was obtained at 159,000× *g* for 5 h, with shorter centrifugation times (3 h) still yielding acceptable results [82].

For influenza virosome production, researchers have successfully removed nucleocapsids using ultracentrifugation followed by dialysis with DCPC as a solubilizing agent. This sequential process not only facilitated the removal of nucleocapsids but also enabled efficient virosome reconstitution, preserving membrane integrity and glycoprotein functionality [83].

The choice of detergent plays a vital role in maintaining the structural integrity of viral proteins during virosome preparation. In particular, appropriate detergents help prevent protein aggregation and preserve the conformation of surface glycoproteins essential for immune recognition. For example, a study on NDV-virosome production demonstrated that using DHPC for viral membrane solubilization enabled efficient nucleocapsid removal by ultracentrifugation while retaining native viral glycoproteins. This optimized approach ensured high immunogenicity and protective efficacy in vaccinated subjects [81].

Another study highlighted the importance of sucrose gradient ultracentrifugation in nucleocapsid separation, enabling a cleaner and more efficient purification of viral proteins [84]. This technique is particularly useful for virosome-based vaccine production, ensuring that non-infectious particles are obtained while retaining functional surface proteins. The efficiency of nucleocapsid removal depends on the use of optimal centrifugation speeds and the appropriate selection of solubilizing detergents to prevent aggregation of viral proteins [83,85].

#### 3.2.3. Reconstitution into Lipid Bilayer

After the removal of the nucleocapsid, the reassembly of viral envelope proteins and lipids into a functional virosome structure is a critical step in virosome production. This process ensures that virosomes retain their structural integrity and functional properties, such as membrane fusion activity and antigen presentation [83,85].

The reconstitution of virosomes involves several key steps, including detergent removal, membrane reformation, and purification, to obtain functional virosome particles.

##### Detergent Removal and Phospholipid Bilayer Formation

Detergent removal is essential to allow the spontaneous formation of phospholipid bilayers, reconstituting the viral envelope in a way that preserves glycoprotein function. Various methods are employed, including dialysis, gel filtration, and Bio-Beads SM-2 treatment [86].

A study demonstrated that the use of a short-chain phospholipid, DCPC, significantly improves the efficiency of detergent removal and virosome formation [83]. Triton X-100 is commonly used for initial solubilization, but it must be carefully removed to avoid residual detergent molecules disrupting the virosome structure [78].

##### Purification by Sucrose Density Gradient Ultracentrifugation

Properly formed virosomes need to be separated from non-incorporated components, including free viral proteins and lipids. This is typically achieved through equilibrium density gradient ultracentrifugation, which isolates intact virosomes based on their buoyant density [13].

Studies on vesicular stomatitis virus (VSV) have shown that virosomes produced through octylglucoside solubilization followed by sucrose gradient ultracentrifugation exhibit fusion activity comparable to native viral envelopes [87].

##### Retention of Fusogenic Activity

The biological function of virosomes depends on their ability to fuse with target cell membranes, a process mediated by viral glycoproteins such as HA and NA [64]. To ensure fusogenic activity, reconstitution methods must prevent glycoprotein denaturation and maintain their proper orientation within the bilayer [66].

A study on Sendai virus envelopes using CHAPS instead of Triton X-100 demonstrated that detergent removal allowed the formation of highly fusogenic virosomes while preserving their functional integrity [88]. Reconstituted influenza virosomes have been shown to maintain pH-dependent membrane fusion properties, crucial for effective antigen delivery in vaccine formulations [89].

### 3.3. Mechanism of Action of Virosomes

Since virosomes consist of a lipid bilayer embedded with viral glycoproteins but lack genetic material, rendering them non-infectious, virosomes can mimic the fusion and cell entry mechanisms of native viruses while delivering encapsulated therapeutic agents or antigens to target cells [89].

Upon administration, virosomes are recognized by APCs such as DCs and macrophages. The viral glycoproteins embedded in the virosomal membrane facilitate receptor-mediated endocytosis, leading to internalization by APCs [66].

**Figure 3:**
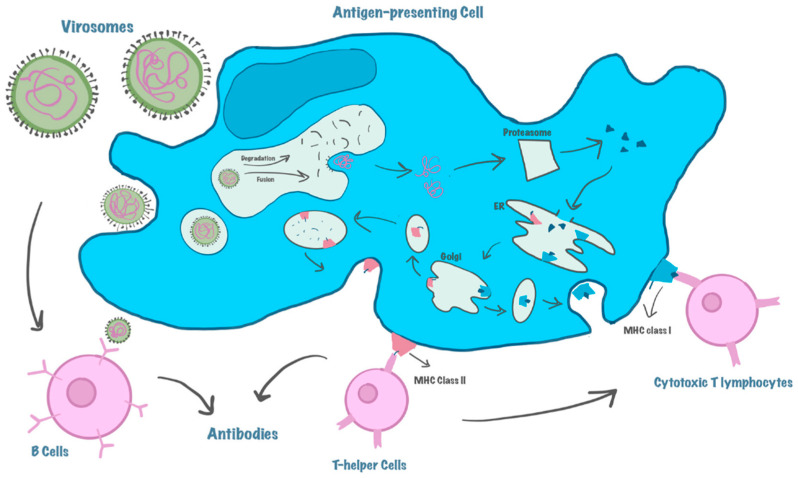
General mechanism of action of virosomes which demonstrates how a virosome interacts with cells which present antigens and triggers an immune response [64].

Virosomes induce a strong immune response by displaying viral antigens in their native conformation (Figure 3) [13]. The lipid bilayer structure ensures antigen stability while maintaining the ability to activate CD4^+^ and T-cell (CD8^+^) responses [64]. The presence of fusion proteins in virosomes allows them to deliver encapsulated macromolecules directly to the cytosol of target cells, bypassing degradation in lysosomes [64]. This property is particularly advantageous for vaccines aimed at eliciting a cytotoxic T lymphocyte (CTL) response, as seen in virosome-based formulations for influenza and hepatitis A vaccines [90].

Upon encountering a target cell, virosomes bind to specific receptors on the cell membrane via surface glycoproteins, commonly HA in influenza-derived virosomes. This interaction facilitates clathrin-mediated endocytosis, leading to internalization into early endosomes [91,92,93].

As endosomes acidify, HA undergoes a pH-dependent conformational change that promotes fusion between the virosomal membrane and the endosomal membrane. This fusion results in content release directly into the cytoplasm, bypassing lysosomal degradation, a key advantage for delivering sensitive drugs or antigens [13].

Fusion facilitates the delivery of encapsulated materials, such as protein antigens or therapeutic molecules, into the cytosol. In APCs, this enables MHC class I presentation and triggers cytotoxic CTL responses, essential for anti-viral or anti-tumor immunity [94]. Once inside the tumoral cell, virosomes fuse with the endosomal membrane, allowing the release of their cargo into the cytoplasm. This mechanism ensures efficient antigen processing and presentation via both MHC class I and II pathways, thereby stimulating both humoral and cell-mediated immune responses [93,94].

The fusion mechanism is solely mediated by HA. At low endosomal pH, HA exposes its hydrophobic fusion peptide, which inserts into the endosomal membrane, pulling both membranes together until fusion occurs. Synthetic virosomes recapitulate this mechanism nearly identically to native influenza viruses [95]. This pH-dependent fusion mechanism, enabled by HA’s structural shift, mimics the native viral entry process and is essential for efficient intracellular delivery of macromolecules, including DNA, siRNA, proteins, and peptides. Unlike many nanoparticles, virosomes avoid degradation in lysosomes due to their timely escape into the cytoplasm, enhancing the bioavailability of their cargo. Their mimicry of viral fusion pathways also enhances cellular uptake and immunogenicity, making them ideal carriers for gene therapy, vaccine delivery, and targeted cancer therapeutics [64].

Moreover, recent innovations such as HA-specific virosomes and bioengineered systems like magnetically guided virosomes are expanding the capabilities of this platform for targeted delivery, including to the lungs or tumor tissues [96].

In addition to vaccines, virosomes function as targeted drug delivery systems. They can encapsulate nucleic acids, proteins, or small-molecule drugs, protecting them from degradation while enabling controlled release at the desired site. Their biocompatibility, biodegradability, and non-toxicity make them an ideal nanocarrier system for cancer immunotherapy, gene therapy, and precision medicine [64].

## 4. Applications and Case Studies for the Production of Vaccines Against Different Diseases

### 4.1. Coronaviruses Vaccines

VLPs and virosomes have emerged as promising vaccine platforms for combating coronaviruses, particularly due to their structural mimicry of native viruses while lacking replicative capacity. These nanoparticle-based systems allow for the safe and effective delivery of antigens and have shown strong immunogenicity in both preclinical and clinical settings [97].

#### 4.1.1. VLPs

Recent advancements in expression technologies have enabled the production of SARS-CoV-2 VLPs using diverse systems such as baculovirus-insect cells, mammalian cells, yeast, and plant-based platforms.

##### Insect Cell-Based Expression Systems

**Figure 4:**
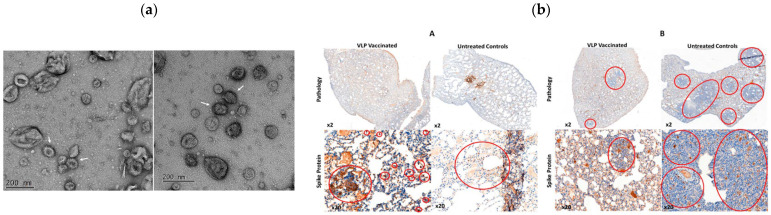
(**a**) Transmission electron microscopy was used to visualize purified SARS-CoV-2 VLPs obtained from insect cell cultures. To prepare samples, the VLP-containing fractions were first diluted to minimize residual sucrose, and then re-concentrated and adsorbed onto carbon-coated grids. The grids were negatively stained with 2% uranyl acetate to enhance structural contrast. Many of these structures displayed distinctive fringe-like projections along their surfaces, which are characteristic of coronavirus spike proteins. Two representative micrographs of the same adsorbed sample illustrate this heterogeneous population of spherical vesicles and spiked envelopes. (**b**) Histological analysis of lung tissue collected from animals euthanized at 2 days (**A**) and 10 days (**B**) after live virus challenge. The (**upper**) panels show hematoxylin and eosin (H&E)-stained sections, highlighting areas of vacuolization and eosinophilic infiltration within the lung parenchyma. The (**lower**) panels present immunohistochemical staining using an anti-spike (S) protein antibody, where brown staining marks the presence of viral antigen. Together, these images illustrate the progression of viral pathology and antigen localization over the course of infection [98].

Sullivan et al. generated SARS-CoV-2 VLPs by co-expressing spike (S), membrane (M), and envelope (E) proteins in insect cells using a single recombinant baculovirus. These VLPs induced potent neutralizing antibodies and protected Syrian hamsters against B.1.1.7 variant challenge by significantly reducing viral load and lung pathology (Figure 4) [98]. A two-component nanoparticle VLP vaccine using baculovirus-expressed S1 protein conjugated to AP205 phage particles achieved strong neutralization of the Wuhan and UK variants, highlighting the value of modular VLP design [99].

##### Plant-Based Expression Systems

Using a plant-based expression system, Lemmer et al. produced Beta variant-specific SARS-CoV-2 VLPs in *Nicotiana benthamiana*. These particles, formulated with adjuvants, provided broad neutralization against Beta, Delta, and Omicron variants in hamsters, supporting their application as versatile pan-sarbecovirus vaccine platforms [100].

##### Mammalian Cell-Based Expression Systems

**Figure 5:**
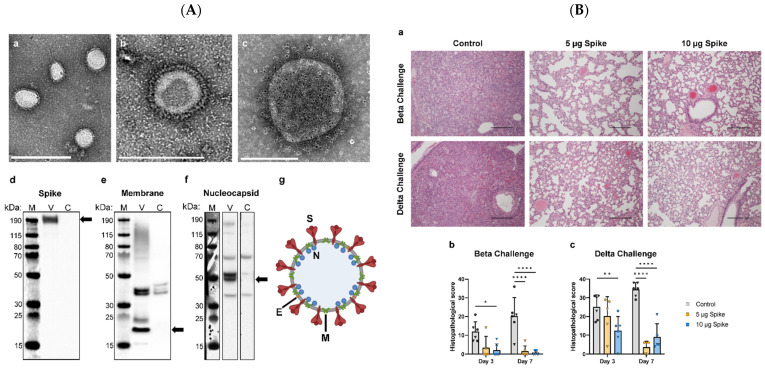
(**A**) (**a**,**b**) Electron micrographs of purified SARS-CoV-2 VLPs, negatively stained with phosphotungstic acid, reveal uniform, spherical particles resembling native virions. Scale bars correspond to 500 nm in (**a**) and 200 nm in (**b**). (**c**) Immunogold labeling using an anti-spike primary antibody and a gold-conjugated secondary antibody highlights spike protein localization on the VLP surface. The gold particles, visible as ~6 nm black dots, clearly outline the envelope’s spike distribution. Scale bar: 200 nm. (**d**–**f**) Western blot analysis of purified VLPs (V) compared with mock-transfected cell lysates (C) used as negative controls. Blots were probed with antibodies specific for (**d**) spike, (**e**) membrane, and (**f**) nucleocapsid proteins. Molecular weight markers (M) are indicated to the left, and arrows identify the target protein bands. (**g**) The schematic illustrates the structural composition of the SARS-CoV-2 VLP, which includes the spike (S), envelope (E), membrane (M), and nucleocapsid (N) proteins assembled into a virus-like architecture but lacking any genetic material. (**B**) Lung histopathology at 3 and 7 days post-challenge. (**a**) Representative histological sections of lung tissue from control and vaccinated animals collected 7 days after challenge with either the Beta or Delta variant of SARS-CoV-2. Lungs from unvaccinated control animals display extensive tissue damage, with loss of normal alveolar architecture, dense leukocyte infiltration within alveolar spaces and septa, fibrin deposition, and widespread hypertrophy and hyperplasia of pneumocytes. Areas of hemorrhage are visible throughout the parenchyma, resulting in near-complete loss of open airspaces (left panels). In contrast, lung sections from vaccinated animals show well-preserved tissue structure with minimal or no inflammatory infiltration and open, aerated alveoli (middle and right panels). Scale bars: 400 µm. (**b**,**c**) Quantitative histopathological scores for hamsters challenged with the Beta (**b**) or Delta (**c**) variants. Each symbol represents an individual animal—females (●) and males (▼)—and bars indicate mean ± SD. The dotted line marks the lower detection limit. Statistical significance was assessed using two-way ANOVA with Tukey’s multiple comparisons test (* *p* < 0.05, ** *p* < 0.01, **** *p* < 0.0001) [101].

In mammalian systems, Resch et al. constructed monovalent and bivalent VLPs by expressing all four SARS-CoV-2 structural proteins (Figure 5). These VLPs provided strong protection in hamsters against Beta and Delta variants and showed partial cross-neutralization of Omicron [101].

Xu et al. demonstrated that VLPs formed in Vero E6 cells better maintained the structural stability and antigenic profile of the native virus than those from HEK293T cells, reinforcing the relevance of host selection in VLP production [102].

**Figure 6:**
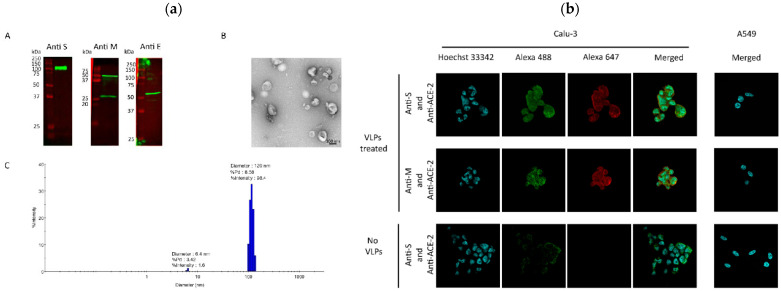
(**a**) Characterization of SARS-CoV-2 VLPs produced from a stable HEK293-F cell pool in a fed-batch stirred-tank bioreactor. (**A**) Western blot analysis confirms the presence of the spike (S), envelope (E), and membrane (M) proteins in purified VLPs obtained using chromatography. Detection of the spike protein indicates its successful incorporation and surface display on the particles. (**B**) Transmission electron microscopy of purified VLPs reveals well-defined, spherical structures consistent with coronavirus morphology. (**C**) Dynamic light scattering analysis shows a narrow particle size distribution, confirming uniformity and stability of the VLP preparations. (**b**) SARS-CoV-2 VLP receptor-binding assay. Calu-3 and A549 cells were incubated either with SARS-CoV-2 VLPs or with mock buffer as a control. Following incubation, VLPs internalized or bound to the cell surface were visualized using antibodies against the membrane (M) and spike (S) proteins, each conjugated to Alexa Fluor^®^ 647 (red). The ACE2 receptor was detected with an anti-ACE2 antibody labeled with Alexa Fluor^®^ 488 (green), while nuclei were counterstained with Hoechst 33342 (blue). In Calu-3 cells, VLP binding colocalized with ACE2 expression, whereas A549 cells lacked detectable ACE2, consistent with their non-permissive phenotype under these conditions [103].

A stable HEK293 cell pool platform enabled scalable VLP production in bioreactors. This platform was scalable up to a 2 L fed-batch bioreactor, producing properly assembled VLPs whose size and morphology resembled authentic virions. The purified VLPs were functional, specifically binding ACE2-expressing cells (Figure 6), demonstrating that the approach enables scalable VLP manufacturing while preserving key functional and structural features [103].

##### Yeast Expression Systems

Yeast-based VLPs were used by Arora et al., employing the D-Crypt™ system to co-express S, M, and E proteins. These VLPs showed high stability and were scalable for industrial vaccine manufacturing [104].

##### Alternative and Hybrid Systems

Nguyen et al. used a BacMam baculovirus vector to co-express SARS-CoV-2 S, M, and E proteins, forming authentic VLPs that elicited spike-specific IgG and neutralizing responses in mice, even in the absence of adjuvants [105].

In another study, Wang et al. explored ALVAC-VLPs engineered using CRISPR/Cas9, which protected hamsters against multiple SARS-CoV-2 variants by activating TLR4 and promoting robust humoral and T-cell immunity [106].

Hassebroek et al. developed HBcAg-based VLPs expressing SARS-CoV-2 B and T cell epitopes, eliciting epitope-specific IgG and moderate protection in transgenic mouse models [107].

Recent omics-guided and computational design strategies have accelerated the development of broad-spectrum VLP vaccines. For example, Zhang et al. engineered a trivalent SARS-CoV-2 VLP vaccine displaying receptor-binding domains (RBDs) from the wild-type, BQ.1.1, and XBB.1 variants. Using structural modeling and immunogenicity profiling, they optimized antigen presentation to enhance cross-neutralization. The resulting construct elicited potent antibody responses against multiple emerging variants, including XBB.1, EG.5, and BA.2.86, demonstrating the potential of integrative design for adaptive VLP vaccine platforms [108].

All SARS-CoV-2 VLP platforms showed strong immunogenicity but differed in yield, fidelity, and scalability. Insect cell systems produced high yields and effective immune protection using baculovirus-expressed structural proteins [98,99], while plant-based systems offered low-cost, scalable production with broad neutralization against multiple variants [103]. Mammalian systems generated VLPs most similar to native virions, providing strong cross-variant protection and suitability for bioreactor scale-up [101,102,103]. Yeast systems ensured stability and industrial scalability, and hybrid systems such as BacMam and ALVAC-VLPs enhanced both humoral and cellular responses [105,106]. Recent omics-guided and computational approaches have further optimized antigen design, as demonstrated by a trivalent RBD VLP inducing potent cross-neutralization against emerging SARS-CoV-2 variants [108]. Overall, mammalian and omics-integrated systems offer the greatest fidelity and adaptability, whereas insect and plant systems provide efficient, cost-effective production.

#### 4.1.2. Virosomes

Influenza virosomes have been widely used as backbones for engineering coronavirus vaccines. By incorporating SARS-CoV or SARS-CoV-2 spike proteins, these constructs retain HA-mediated endosomal fusion, allowing the spike antigen to enter the cytosol and enhance both humoral and cellular immunity [109,110].

Early foundational work demonstrated this principle with influenza-SARS-CoV fusion virosomes, leading to mucosal immunity in murine models [111]. Influenza-based virosomes encapsulating synthetic mRNA encoding SARS-CoV-2 spike protein showed protection in mice, with induction of cytotoxic T cells and neutralizing antibodies. This strategy avoided viral vectors while retaining delivery efficiency [112].

van der Velden et al. developed virosome-based vaccines incorporating the SARS-CoV-2 spike protein (from Wuhan and Beta variants). In murine models, two doses induced potent neutralizing antibody responses and significant mucosal immune activation, demonstrating strong protection upon viral challenge. The Wuhan spike virosome produced broader neutralization across variants, indicating effective mucosal and systemic immunity [113].

**Figure 7:**
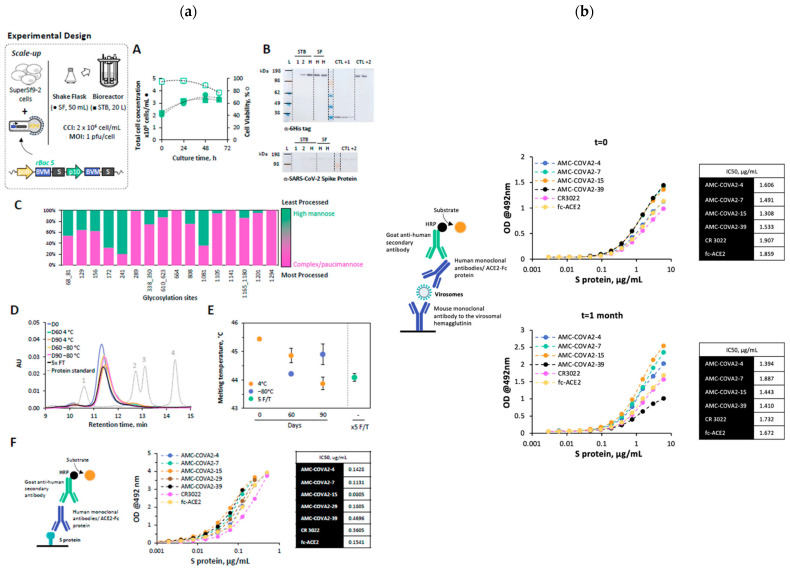
(**a**) Production of the SARS-CoV-2 spike (S) protein was carried out in a 20-L stirred-tank bioreactor (STB). (**A**) Cell growth was monitored following infection, showing typical kinetics over the course of the process. (**B**) Western blot analysis confirmed the presence of the S protein in supernatant samples collected at 1, 2, and 2.6 days post-infection (63 h). Both a mouse anti-6× His antibody and a human anti–SARS-CoV-2 spike antibody detected the expected ~140 kDa monomeric S band. Samples from the STB and shake-flask (SF) cultures were compared, with in-house purified His-tagged spike proteins used as positive controls (0.2–0.05 µg and 1–0.5 ng, respectively). (**C**) Glycosylation patterns of the purified protein were characterized by mass spectrometry. Site-specific analysis revealed both high-mannose structures (M9–M5; Man9GlcNAc2–Man5GlcNAc2) and complex/paucimannose glycans, indicating correct processing and folding. (**D**) Size-exclusion chromatography (HPLC-SEC) assessed protein stability after storage at various temperatures and after five freeze–thaw cycles. The retention profile remained consistent with a predominantly trimeric species, confirming structural stability. (**E**) Thermal stability, measured by differential scanning fluorimetry, showed a reproducible melting profile across triplicate runs (*n* = 3), reflecting good conformational integrity. (**F**) Functional binding assays demonstrated that the recombinant S protein maintained strong affinity for the ACE2 receptor and for neutralizing human antibodies (ACE2-NN-IgGFc, CR3022, and other anti-SARS-CoV-2 antibodies). ELISA results confirmed preserved antigenicity and epitope accessibility following production and purification. (**b**) Binding of antibodies to virosome-associated spike (S) protein was evaluated by ELISA. Virosomes captured on plates pre-coated with anti-hemagglutinin antibodies were incubated with a panel of non-overlapping human neutralizing antibodies targeting distinct epitopes within the receptor-binding domain (RBD) of the spike protein, as well as with an ACE2-Fc fusion protein to assess receptor interaction. Detection was performed using goat anti-human HRP conjugate. Panel (**A**) shows antibody and ACE2 binding at the time of virosome production, while Panel (**B**) depicts the same analysis after one month of storage at 4 °C. Comparable binding profiles across both time points indicate that the S protein retained its structural integrity, antigenicity, and functional receptor-binding capacity during storage [114].

A cutting-edge approach used insect cells (IC-BEVS system) to express pre-fusion stabilized SARS-CoV-2 spike protein. The insect cell-derived spike protein maintained ACE2 receptor binding, antigenicity, and recognition by neutralizing antibodies, both as purified protein and when displayed on virosomes (Figure 7). The virosomes remained stable for over one month at 4 °C, confirming their robustness and manufacturability. This work established IC-BEVS as a scalable, efficient platform for producing complex glycoproteins for virosomal vaccine applications [114].

More recently, multivalent virosomes presenting envelope proteins alongside other viral antigens such as hepatitis C or SARS-CoV-2 have been developed to explore cross-pathogen protection and multiplexed immunization strategies. These bivalent or trivalent constructs are currently in preclinical evaluation [115].

These advances illustrate that virosome-based coronavirus vaccines can elicit robust neutralizing and mucosal immune responses, maintain antigenic stability during production, and offer flexible formulation routes for systemic or mucosal immunization. As such, virosomes represent a next-generation vaccine platform adaptable for pan-β-coronavirus protection, with potential scalability for global deployment [116].

Influenza-based virosomes incorporating SARS-CoV or SARS-CoV-2 spike proteins effectively utilized HA-mediated fusion to enhance antigen delivery and induce both humoral and cellular immune responses [109,110,111]. mRNA-loaded virosomes encoding the SARS-CoV-2 spike achieved potent cytotoxic T-cell and neutralizing antibody responses, combining efficient delivery with viral vector–free design [112]. Spike protein-decorated virosomes, such as those developed by van der Velden et al., provided broad mucosal and systemic protection against Wuhan and Beta variants, highlighting strong cross-variant immunity [113]. The IC-BEVS insect cell system enabled large-scale production of prefusion-stabilized spike proteins for virosome display, ensuring high antigen stability and manufacturability [114]. Furthermore, multivalent virosomes presenting combinations of SARS-CoV-2 and other viral antigens, including hepatitis C envelope proteins, demonstrated potential for multiplexed and cross-pathogen vaccination [115]. Overall, SARS-CoV-2 virosome technologies offer robust immunity, scalable production, and adaptable formulations suitable for next-generation pan-β-coronavirus vaccine development [116].

### 4.2. Vaccines Against Influenza Viruses

#### 4.2.1. VLPs

##### Insect Cell-Based Expression Systems

A widely used method for producing VLPs uses Sf9 insect cells. Galarza et al. demonstrated that influenza VLPs composed of HA and matrix protein 1 (M1), produced in Sf9 insect cells using a recombinant baculovirus system, were highly immunogenic and conferred complete protection in mice. Specifically, mice vaccinated with ~1 µg HA of VLPs (intramuscularly or intranasally) showed strong antibody titers and 100% survival following lethal challenge with influenza A/Hong Kong/68 (H3N2) virus. Intranasal vaccination also presented stronger mucosal immune responses [117]. In a more recent study, Ren et al. showed that H7N9 VLPs (HA, NA, M1) produced in Sf9 cells provided 100% protection in mice when administered by both intranasal and intramuscular routes, reinforcing the success of the same expression system in other influenza subtypes [118].

##### Plant-Based Expression Systems

Tobacco-derived VLPs have shown similar assembly pathways and immunogenicity to mammalian-produced particles, with no hypersensitivity observed in human trials [119]. H3N2-based VLPs protected dogs from wild-type infection, particularly when combined with Montanide ISA adjuvants [120].

##### Mammalian Cell-Based Expression Systems

HEK-293 and CHO cells enable human-like glycosylation. VLPs produced in HEK-293 cells expressing H1N1 HA and NA conferred full protection and induced strong mucosal immunity in mice [121].

New VLPs produced using advanced mammalian systems (e.g., 293F and Vero cells) and optimized purification techniques are being developed for multivalent and universal vaccine strategies [47].

##### Yeast Expression Systems

H1N1 and H3N2 VLPs expressed in *Saccharomyces cerevisiae* provided potent neutralizing responses in preclinical models [122].

##### Avian Expression Systems

Chickens that were vaccinated with a dual-clade H5 VLP showed complete survival and reduced viral shedding, with scalable production in low-cost systems [123]. VLPs which display conserved domains like HA stalks and M2e have been shown to induce cross-protection against multiple influenza subtypes [124].

##### Multivalent and Next-Generation VLPs

**Figure 8:**
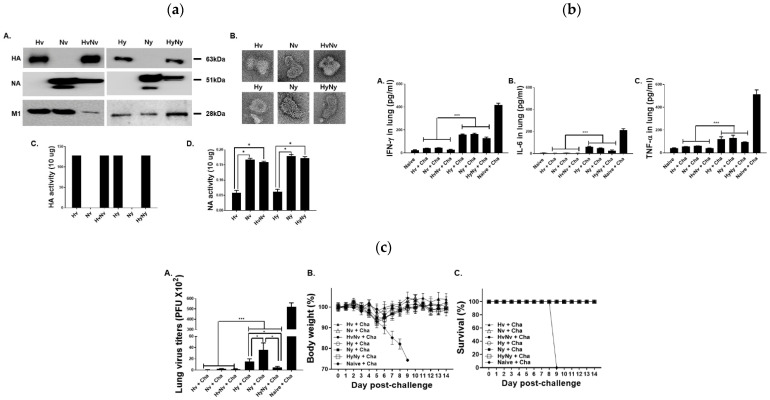
(**a**) Characterization of the influenza B VLP vaccines involved several complementary assays. (**A**) Western blot analysis confirmed the expression of HA, NA, and M1 proteins using both monoclonal and polyclonal antibodies, verifying successful assembly of the particles. (**B**) The morphology of the purified VLPs was examined by transmission electron microscopy after negative staining, revealing spherical particles resembling native virions. (**C**) Hemagglutination activity was tested using chicken red blood cells to confirm the functional integrity of the HA protein. (**D**) Enzymatic activity of neuraminidase was measured with the Amplex Red assay, demonstrating that the VLPs retained catalytic function. All quantitative data are shown as mean ± SD, and statistical differences between groups are indicated by asterisks (* *p* < 0.05). (**b**) Pro-inflammatory responses in the lungs were analyzed following challenge with influenza B virus. At 4 days post-infection (dpi) with the B/Colorado/06/2017 strain, cytokine levels of IFN-γ (**A**), IL-6 (**B**), and TNF-α (**C**) were quantified in lung homogenates. The Naïve + Cha group represents unvaccinated mice exposed to a lethal viral dose, serving as the infection control. Cytokine concentrations were measured to evaluate inflammation severity and immune modulation after vaccination. Data are presented as mean ± SD, with statistical differences between groups indicated by asterisks (*** *p* < 0.001). (**c**) Lung viral loads, body weight, and survival were monitored to assess protection against lethal influenza B challenge. BALB/c mice were infected with a lethal dose of influenza B virus, and vaccine efficacy was evaluated by comparing lung virus titers (**A**), body weight changes (**B**), and survival rates (**C**) across groups. The Naïve + Cha group represents unvaccinated mice exposed to the same viral challenge, serving as the control for disease progression. Data are shown as mean ± SD, with statistically significant differences between groups indicated by asterisks (* *p* < 0.05, *** *p* < 0.001) [125].

A research group developed influenza B VLP vaccines expressing HA and NA from both the B/Victoria (B/Washington/02/2019-like) and B/Yamagata (B/Phuket/3073/2013-like) lineages. Immunising the mice with either lineage-derived or dual VLP formulations induced robust antibody titers (HI and IgG) and mucosal IgA responses. Following the challenge with mismatched B/Colorado/06/2017 (Victoria lineage) virus, vaccinated mice showed 100% survival, minimal body weight loss, and drastically reduced lung viral loads. The protection correlated with enhanced CD4^+^ and CD8^+^ T-cell activation, germinal center B-cell responses, and cytokine modulation in the lungs, confirming the development of both humoral and cellular immunity (Figure 8) [125].

Incorporation of GM-CSF and flagellin in H5N1 VLPs improved humoral and mucosal responses, suggesting potential for cross-subtype protection [126]. Insect cell-based systems (Sf9) remain the most established, producing HA/M1 or HA/NA/M1 VLPs that confer full protection and robust mucosal immunity, validating their efficiency and reproducibility across influenza subtypes [117,118]. Plant-based systems achieve similar antigen assembly and safety to mammalian-derived VLPs, demonstrating efficacy in both animal and veterinary models while offering cost-effective scalability [119,120]. Mammalian expression platforms (HEK-293, CHO, 293F, Vero) provide human-like glycosylation and high structural fidelity, yielding strong systemic and mucosal responses suitable for multivalent and universal vaccine strategies [47,121]. Yeast and avian systems further contribute economical, stable production routes and generate cross-protective immunity across influenza subtypes [122,123,124]. Emerging multivalent VLP formulations combining influenza A and B antigens induced broad and balanced humoral and cellular responses, including enhanced CD4^+^ and CD8^+^ activation, robust IgA/IgG titers, and complete survival in lethal challenge models [125]. Incorporation of immunostimulatory elements such as GM-CSF and flagellin further improved both mucosal and systemic immunity, indicating promise for cross-subtype protection and next-generation universal influenza vaccines [126].

#### 4.2.2. Virosomes

One of the earliest clinical examples of licensed virosomal influenza vaccines is Inflexal^®^ V, which incorporated HA from seasonal strains and was shown to elicit robust humoral responses in pediatric and elderly populations. These vaccines were well tolerated and induced strong neutralizing antibody titers with excellent safety profiles [127].

The combination of MF59 adjuvants with virosomes has also been studied. These co-formulations enhanced antigen uptake by DCs, boosting CD8^+^ T-cell responses and improving efficacy against drifted H3N2 strains in older adults [128].

Intranasal delivery of virosomal vaccines offers mucosal immunity, a key defense for respiratory pathogens. Studies using heat-labile enterotoxin (HLT) as an adjuvant demonstrated strong secretory IgA and systemic IgG responses in ferrets and humans, showing promise for needle-free immunization strategies [129].

CpG-ODN adjuvanted virosomes have been shown to significantly reduce viral shedding and improve HI titers in avian influenza models, suggesting viability for pandemic influenza control in poultry and possibly humans [130].

A novel monophosphoryl lipid A-adjuvanted virosome formulation included Ni-chelating lipids for attaching conserved nucleoprotein (NP) antigens. This platform induced CTL responses and protection in mice, though excessive CTL priming raised concerns about disease exacerbation in some contexts [131].

Virosome-based DNA vaccines have been explored for delivering viral genes such as those from mumps and influenza viruses, inducing both systemic and mucosal immunity when administered intranasally [132].

A comparative study between MF59-adjuvanted and virosomal subunit vaccines showed that both were effective in reducing hospitalizations due to influenza in adults aged 65+, suggesting comparable real-world effectiveness [133].

Virosome–nanoparticle hybrids, combining lipid membranes with polymeric cores, are a new innovation designed to improve thermostability and antigen release control, thus enhancing shelf-life and long-term immunity [134].

The licensed Inflexal^®^ V vaccine incorporating seasonal HA antigens achieved strong neutralizing antibody responses and outstanding safety in pediatric and elderly populations [127]. The addition of adjuvants such as MF59, CpG-ODN, and monophosphoryl lipid A enhanced antigen uptake, T-cell activation, and cross-strain protection in both human and animal models [128,129,130,131]. Intranasal administration, particularly when combined with adjuvants like HLT, effectively induced mucosal IgA and systemic IgG responses, supporting needle-free immunization [129,132]. Comparative studies confirmed that MF59-adjuvanted and virosomal subunit vaccines performed similarly in reducing influenza-related hospitalizations among older adults [133]. Hybrid virosome–nanoparticle formulations improved thermostability and controlled antigen release, extending shelf-life and the durability of immune protection [134].

Together, these developments highlight the adaptability and promise of virosomal platforms for next-generation influenza vaccines, especially in combination with advanced adjuvants, novel delivery routes, and multi-antigen targeting.

### 4.3. Newcastle Disease Virus

NDV is a highly contagious avian pathogen that poses major economic and biosecurity challenges to the poultry industry worldwide. Despite the availability of conventional live-attenuated and inactivated vaccines, recurrent outbreaks continue to occur, largely due to antigenic variability among NDV genotypes and limitations in differentiating infected from vaccinated animals (DIVA) [135,136].

#### 4.3.1. VLPs

VLP-based vaccines against NDV have been developed using diverse expression systems, each offering distinct advantages in terms of scalability, immunogenicity, and production feasibility. These systems include insect cell-, avian cell-, and plant-based platforms, as well as innovative chimeric approaches that combine NDV antigens with those of other viruses such as avian influenza.

Table 2 summarizes representative NDV VLP constructs, outlining their expression systems, antigenic compositions, immunization outcomes, and unique advantages. Together, these studies demonstrate the versatility of NDV VLP technology for developing safe and effective vaccines adaptable to both veterinary and potentially human applications.

All NDV VLP production systems demonstrated strong protective efficacy, yet they differ in scalability, immune profile, and technical suitability. Insect cell-based systems achieved high yields and incorporated heterologous matrix proteins, offering robust humoral responses and DIVA compatibility, which are advantageous for industrial vaccine manufacturing [137]. Avian cell-derived VLPs most closely replicated native virion architecture and elicited balanced humoral and cellular immunity, making them ideal for immunogenicity studies and multivalent antigen display [138,139]. Plant-based systems provided a rapid, low-cost, and easily scalable alternative, producing VLPs with strong antibody responses suitable for large-scale deployment in endemic regions [140]. Finally, chimeric NDV–H5N1 VLPs extended the platform’s utility by conferring dual protection against two major avian pathogens while retaining DIVA functionality [141]. Overall, insect and avian systems offer high fidelity and immune potency, whereas plant-based expression provides the best scalability and production flexibility.

#### 4.3.2. Virosomes

Incorporating immunostimulatory adjuvants such as CpG-ODN into NDV virosomes markedly enhances innate immunity and protection in broilers and layers, increasing HI titers and reducing viral shedding [142]. CpG-ODN-adjuvanted virosomes strengthen both mucosal and systemic responses, while intranasal and ocular delivery offers an effective, needle-free approach for mass poultry immunization. Mucosal administration induces strong local IgA and systemic IgY antibodies, ensuring robust protection and practical application in farm settings [142].

Ni-chelating virosomes conjugated with conserved internal NDV antigens, such as the nucleocapsid (NP) protein, have been used to stimulate cell-mediated immunity in addition to humoral responses. These approaches show promise for improving cross-protection across NDV genotypes, especially in endemic regions. Virosomes presenting conserved NDV proteins enhance cross-genotype immunity in chickens [143].

DNA-based NDV vaccines delivered via virosomes, which encapsulate plasmids encoding the F or HN antigens, have shown strong immunogenicity in poultry. Intranasal administration induced both mucosal and systemic immune responses, providing effective protection against virulent NDV challenge in chickens [144]. Comparative studies indicate that while live-attenuated vaccines induce faster immunity, virosomal DNA vaccines offer greater safety, stability, and suitability for regions with limited cold chain infrastructure and emerging virulent strains [145].

Hybrid virosome–polymer nanoparticle systems are being investigated for NDV to improve thermostability and antigen release kinetics, offering enhanced shelf-life and logistical advantages for vaccination programs in rural or resource-limited areas [146].

NDV virosomal platforms show strong immunogenicity, safety, and scalability for poultry vaccination. CpG-ODN-adjuvanted virosomes enhanced innate responses, increased HI titers, and reduced viral shedding via intranasal or ocular delivery, providing an effective needle-free option [142]. Ni-chelating virosomes displaying conserved NP antigens improved cross-genotype protection through combined humoral and cellular immunity [143]. DNA-loaded virosomes encoding F or HN antigens induced dual systemic and mucosal responses, offering safe, stable alternatives to live vaccines, particularly in low-resource regions [144,145]. Hybrid virosome–nanoparticle systems improved thermostability and antigen release for extended shelf-life [146].

Together, these developments emphasize the versatility and growing potential of virosomal technologies for NDV control, offering safer, more stable, and more effective vaccination options for the global poultry industry.

### 4.4. Malaria

#### 4.4.1. VLPs

Recent breakthroughs have focused on VLPs presenting epitopes from the *Plasmodium falciparum* CSP, a major surface antigen during the sporozoite stage. A recent study demonstrated that Qβ and MS2 bacteriophage-derived VLPs displaying a key CSP epitope (recognized by the potent monoclonal antibody L9) induced sterilizing immunity in mice, outperforming the licensed RTS,S/AS01 vaccine in some models. These vaccines presented strong anti-CSP antibody titers and prevented *Plasmodium* liver-stage infection [147].

Blood-stage malaria vaccines aim to prevent the parasite’s replication in red blood cells. An influenza VLP vaccine co-expressing *Plasmodium* berghei AMA1 and microneme-associated antigen elicited robust IgG2a, CD4^+^/CD8^+^ T-cell, and germinal center B-cell responses, resulting in reduced parasitemia and improved survival in mice [148].

Transmission-blocking vaccines aim to prevent *Plasmodium* from completing its life cycle in mosquitoes. A study engineered *Acinetobacter phage* AP205 VLPs conjugated with *Plasmodium falciparum* Pfs47 using the SpyTag/SpyCatcher system. Mice immunized with these particles developed high-affinity antibodies that achieved up to 98% transmission reduction activity in mosquito feeding assays [149].

The RH5.2-VLP vaccine, currently in phase 1 clinical testing, uses a SpyTag/SpyCatcher-conjugated VLP system to display a stabilized form of *Plasmodium falciparum* RH5 (a key erythrocyte invasion antigen). This formulation significantly enhanced antibody quantity and growth-inhibitory potency compared to soluble RH5.1 protein vaccines [150]. Malaria VLP vaccines show strong potential across multiple Plasmodium life stages by eliciting robust antibody and T-cell responses. Sporozoite-stage VLPs (Qβ and MS2 bacteriophage-based) displaying the P. falciparum CSP epitope achieved sterilizing immunity and outperformed RTS,S/AS01 in mice [147]. Blood-stage influenza VLPs co-expressing AMA1 and microneme antigens reduced parasitemia and improved survival [148], while transmission-blocking AP205 VLPs presenting Pfs47 induced high-affinity antibodies that cut mosquito transmission by up to 98% [149]. The RH5.2-VLP vaccine, now in phase 1 trials, further enhanced antibody titers and growth inhibition compared with soluble antigens, confirming the clinical promise of VLP-based malaria immunization [150].

#### 4.4.2. Virosomes

One of the earliest clinical studies on virosomal malaria vaccines featured formulations incorporating the *Plasmodium falciparum* circumsporozoite protein (CSP), particularly the NANP repeat region, presented on influenza-derived virosomes. One such candidate, PEV3B, demonstrated safety and immunogenicity in children and adults, inducing strong antibody responses and partial protection in field trials conducted in endemic regions [151].

The inclusion of immunostimulatory adjuvants such as synthetic TLR9 agonists (CpG-ODN) in virosomal malaria vaccines has been shown to significantly enhance Th1-biased immune responses and antibody avidity. These co-formulations increased protection against sporozoite challenge in preclinical models and are under evaluation for multi-antigen inclusion strategies. CpG-enhanced virosomal malaria vaccines promote robust and durable immunity in rodent challenge models [152].

Mucosal delivery of malaria virosomes has been explored as a route to generate both systemic and local immunity at entry sites of sporozoites. Intranasal administration with mucosal adjuvants such as heat-labile enterotoxin (HLT) has been shown to induce mucosal IgA and serum IgG responses, opening possibilities for needle-free vaccination in mass immunization campaigns. Intranasal virosomal malaria vaccines elicit mucosal and systemic immunity against *Plasmodium falciparum* [153].

Comparative studies between virosomal malaria vaccines and RTS,S (the leading subunit vaccine) suggest that while RTS,S remains more advanced in licensure, virosomal platforms offer increased flexibility in antigen design and adjuvant pairing, potentially enhancing cross-strain protection and long-term efficacy [154].

The PEV3B candidate, featuring Plasmodium falciparum CSP epitopes on influenza-derived virosomes, proved safe and immunogenic in both children and adults, inducing strong antibody responses and partial field protection in endemic regions [151]. The addition of CpG-ODN adjuvants enhanced Th1-biased immunity and antibody avidity, increasing protection in preclinical challenge models [152]. Intranasal delivery of virosomal malaria vaccines, particularly with mucosal adjuvants like HLT, elicited strong IgA and IgG responses, supporting needle-free immunization strategies [153]. Comparative studies indicate that while RTS,S remains the benchmark subunit vaccine, virosomal systems offer greater flexibility for antigen design and adjuvant integration, potentially improving cross-strain coverage and long-term efficacy [154].

Collectively, these advances highlight the adaptability of virosomal platforms in malaria vaccine development, enabling targeted delivery, adjuvant synergy, and multistage antigen presentation; all these are critical for tackling a complex parasite with diverse life cycle stages and immune evasion strategies.

### 4.5. VLPs as Platforms for Hepatitis Vaccines

Recent advances in vaccine biotechnology have reignited interest in VLPs as versatile platforms for the development of next-generation hepatitis vaccines.

**Figure 9:**
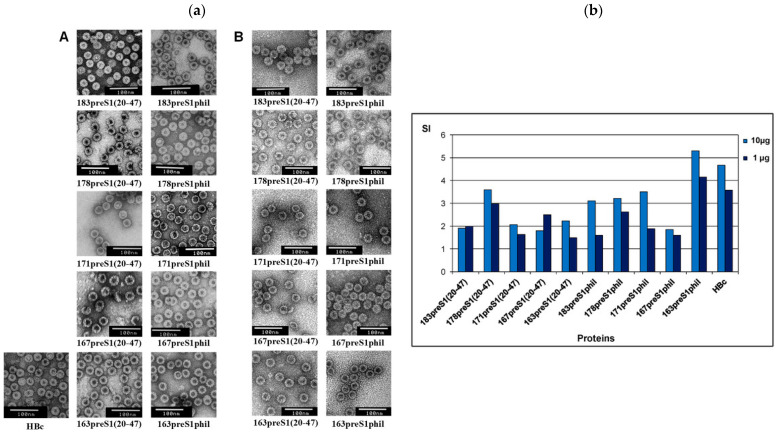
(**a**) EM of HBc-preS1 VLP preparations. (**A**) Original preparations (years 2016–2018). (**B**) The same samples in the year 2021. Scale bar, 100 nm. (**b**) T-cell proliferation in splenocytes of BALB/c mice immunized with HBc-preS1 VLPs. Stimulation indexes (SI) were calculated after stimulation with HBc183 (1 µg and 10 µg) on day 42 after the first immunization. Types of VLPs used for immunization are shown under the bars [155].

A recent report described five variants of the hepatitis B core protein (HBc), both full-length and C-terminally truncated, by inserting short (amino acids 20–47) and long (amino acids 12–60 + 89–119) fragments of the preS1 region. These modified proteins successfully assembled into VLPs and were evaluated for their production efficiency and immune-stimulating properties. All HBc-preS1 constructs expressed strongly, yielding 10–20 mg of highly purified VLPs/gram of biomass after gel-filtration and ion-exchange purification, reaching about 90% purity. When tested in BALB/c mice, the chimeric VLPs triggered robust anti-preS1 antibody production and strong T-cell proliferation following stimulation with HBc antigen (Figure 9). The study also showed that oligonucleotide ODN 1668 could be selectively incorporated into the engineered HBc-preS1 VLPs, further enhancing their potential as versatile vaccine platforms [155].

Computational modeling has increasingly guided the structure-based design of HBV VLPs. By inserting antibody-binding fragments of the hepatitis B surface antigen (HBsAg) into the major immunodominant region of the core antigen (HBcAg), researchers created a chimeric VLP aimed at eliciting both humoral and cellular immunity. In silico analyses confirmed the construct’s stability, antigenicity, and immunogenic potential, while molecular docking demonstrated strong predicted binding to B-cell antibodies, indicating a capacity to induce robust immune responses [156].

Altogether, these advancements highlight the significant potential of VLP platforms in both preventive and therapeutic hepatitis vaccine development. Their versatility in antigen presentation, delivery methods, and adjuvant compatibility positions them as strong candidates for future global hepatitis control strategies.

Also, it is important to note that there are very few new peer-reviewed reports specifically on virosomal hepatitis vaccines (e.g., virosome carriers with hepatitis antigens) published in the recent years. Most licensed virosomal hepatitis vaccines (like Epaxal^®^ for hepatitis A) remain older, but recent reviews and research discuss virosome platforms and their potential for hepatitis vaccine development [13,63,64].

### 4.6. Respiratory Syncytial Virus

Respiratory syncytial virus (RSV) has long been a challenging target for vaccines because of the risk of vaccine-enhanced disease seen with early inactivated formulations.

#### 4.6.1. VLPs

In the case of RSV, VLPs have been studied as a flexible platform to present key viral proteins, especially the fusion (F) protein in its prefusion form (Pre-F) and the attachment (G) protein.

**Figure 10:**
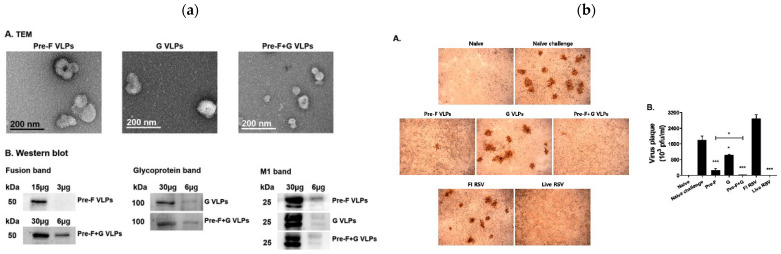
(**a**) VLP characterization. VLPs expressing the RSV Pre-F, G, or both Pre-F and G antigens were visualized by transmission electron microscopy after negative staining (**A**). Influenza M1, RSV fusion, and glycoprotein antigen expressions on the VLPs were determined by Western blots (**B**). (**b**) Lung viral titer after RSV A2 infection. Lung viral titers were determined individually (n = 6) from the lung homogenate. Lung tissues were collected from individual mice 5 days after RSV infection. To confirm the viral plaque formation, DAB substrate was used ((**A**) scale bar, 500 μm). VLP-immunized mice showed significantly lower levels of viral titers than naïve challenged mice ((**B**) * *p* < 0.05, *** *p* < 0.001) [157].

Researchers designed a VLP vaccine expressing both the Pre-F and G proteins using a baculovirus expression system (Figure 10a). The resulting particles were tested in mice and were shown to produce strong RSV-specific immune responses, characterized by elevated antibody titers (Figure 10b) and activation of both CD4^+^ and CD8^+^ T cells. Notably, these VLPs conferred protection against RSV challenge without inducing the excessive eosinophilic inflammation that was seen with early vaccine candidates [157].

Building on these findings, a study further optimized VLP constructs co-expressing the Pre-F and G proteins, including tandem repeat regions to enhance epitope exposure. Immunized mice developed potent neutralizing antibody responses and a Th1-biased cellular immune profile, with higher levels of interferon-γ and RSV-specific CD8^+^ T-cell infiltration in lung tissue. The vaccine reduced viral replication and inflammation following challenge with recombinant RSV, confirming its ability to elicit balanced and effective immunity [158].

More recently, research has shifted toward engineering VLPs that stabilize the prefusion conformation of the F protein. In 2025, Hwang and colleagues developed VLPs displaying RSV F variants designed to preserve critical neutralizing epitopes. These vaccines induced high levels of IgG antibodies against both prefusion and postfusion forms of the F protein and produced robust protection in cotton rat models. Such findings underscore the value of structural biology in guiding RSV VLP design and improving antigen stability [159].

Now, VLP platforms are being applied to create multivalent vaccines that protect against more than one respiratory virus. One of the most advanced examples is IVX-A12, a bivalent VLP vaccine that targets both RSV and human metapneumovirus (hMPV). In a 2025 Phase 1 clinical trial involving adults aged 60–75 years, IVX-A12 was well tolerated and generated strong neutralizing antibody responses against both viruses, marking one of the first VLP-based RSV vaccines to enter human trials. The results suggest that VLPs can serve as flexible scaffolds for co-presenting antigens from multiple pathogens in a single immunization [160].

VLPs co-expressing the prefusion F (Pre-F) and attachment G proteins effectively induced high RSV-specific antibody titers and activated both CD4^+^ and CD8^+^ T cells without triggering the eosinophilic inflammation associated with early vaccine candidates [157]. Optimized Pre-F/G VLPs incorporating tandem repeat regions further enhanced epitope exposure, driving potent neutralizing responses and a Th1-biased immune profile characterized by increased interferon-γ and CD8^+^ T-cell infiltration, leading to reduced viral replication and inflammation [158]. Structural-guided designs have advanced this field further, and prefusion-stabilized F protein VLPs elicited broad IgG responses and conferred strong protection in cotton rat models, underscoring the role of antigen stability in next-generation RSV vaccine efficacy [159].

Translational progress is evident with IVX-A12, a bivalent RSV–hMPV VLP vaccine now in Phase 1 clinical testing. The vaccine was well tolerated in older adults and induced robust neutralizing antibodies against both viruses, highlighting the feasibility of VLPs as modular platforms for multivalent respiratory immunization [160].

#### 4.6.2. Virosomes

One of the most extensively studied virosome strategies for RSV involves the use of monophosphoryl lipid A (MPLA) as an adjuvant incorporated into RSV virosomes. In preclinical animal models, these MPLA-RSV virosomes have shown:

- Strong neutralizing antibody induction: MPLA-adjuvanted virosomes elicited robust virus-neutralizing IgG responses in mice and cotton rats.
- Balanced Th1-biased cellular immunity: Compared with formalin-inactivated RSV, MPLA virosomes induced higher interferon-γ (IFN-γ) production and lower Th2 cytokine responses, which is desirable for avoiding enhanced respiratory disease.
- Protection in challenge models: Animals immunized with RSV-MPLA virosomes cleared live RSV infection with minimal lung pathology and minimal eosinophilic infiltration.

Overall, these findings support MPLA-adjuvanted RSV virosomes as safe and immunogenic vaccine candidates warranting further evaluation toward human use [161].

Some early studies have also explored mucosal administration of RSV virosomes, which is a promising route because RSV infects the respiratory tract. Intranasal virosomes demonstrated immunogenicity in small animal models and helped generate mucosal IgA responses that could contribute to blocking viral entry at the site of infection [162].

In addition to MPLA, lipid-linked peptide adjuvants have been included in RSV virosomes to further enhance immunogenicity. These formulations appear to activate innate immune receptors and drive stronger adaptive responses in animal studies, though they remain largely in early preclinical testing [163].

### 4.7. HIV

#### 4.7.1. VLPs

VLPs are typically built around the self-assembling HIV Gag protein, which forms the structural core of immature HIV particles and can display surface envelope proteins such as gp120 or gp41 trimers in a conformationally native-like context, thereby enhancing the potential to induce both neutralizing antibody and cellular immune responses [164].

Recent preclinical advances have continued to leverage these properties. For example, a novel study described HIV-1 VLPs engineered to maximize antigen density on the particle surface by fusing gp41-derived sequences to Gag, which improved exposure of conserved neutralization epitopes and supported stronger antibody binding compared with lower-density constructs. This strategy reflects a broader effort to design VLPs that present HIV envelope components in ways that better engage B cells and elicit broadly neutralizing antibodies (bNAbs), which remain a central challenge in HIV vaccine design [165].

Recent studies show that mRNA vaccines encoding HIV Env and Gag can form mature VLPs in vivo when co-expressed with a retroviral protease, producing higher neutralizing antibody titers and demonstrating the potential of combining VLP design with mRNA–LNP delivery for enhanced immunogenicity [166].

Beyond particle engineering, recent HIV VLP research has also examined multi-epitope VLP designs. A recent report investigated chimeric VLPs bearing linked linear epitopes from HIV-1 gp120 and gp41 in a modular scaffold, showing successful assembly and induction of both antibody and cellular responses in mouse models, pointing toward the feasibility of epitope-focused VLP vaccines that target conserved regions of the HIV envelope [167].

Reviews of the HIV vaccine field say that VLPs remain a critical component of the diversified vaccine landscape alongside other emerging technologies such as SOSIP trimer scaffolds, nanoparticles, and mRNA constructs. Although early VLP vaccine candidates, for example, a p24-based VLP tested in Phase I/II trials, were safe but poorly immunogenic, progress in antigen design and presentation has renewed interest in the platform for both prophylactic and therapeutic applications [167,168].

Fundamentally, VLPs hold several theoretical advantages for HIV immunization: their particulate nature promotes uptake by antigen-presenting cells and facilitates multivalent display of conformational epitopes; they can engage both B-cell and T-cell arms of immunity; and they can be combined with modern adjuvants or delivery systems to enhance breadth and durability of responses [11].

While no VLP-based HIV vaccine has yet achieved late-stage clinical efficacy, continued innovations in particle design, epitope focus, and integration with contemporary platforms suggest that VLPs remain a versatile and promising avenue toward vaccines capable of eliciting broadly neutralizing antibody and cytotoxic T lymphocyte responses against a highly mutable pathogen like HIV [169].

#### 4.7.2. Virosomes

One of the earliest clinical investigations into virosomal HIV vaccines featured virosomes incorporating gp41 or gp120 envelope glycoproteins, designed to elicit neutralizing antibody responses. These formulations demonstrated favourable safety and tolerability profiles, particularly in early-phase trials involving healthy adults, and succeeded in priming modest humoral responses [170].

The combination of TLR-based adjuvants such as CpG-ODN with virosomal HIV antigens has been shown to significantly enhance dendritic cell maturation and antigen presentation. These co-formulations improved the breadth of CD8^+^ T-cell responses, showing partial protection in non-human primate models against SHIV (Simian–Human Immunodeficiency Virus) challenges. CpG-ODN-adjuvanted virosomes boost cellular immunity and partial protection in simian models of HIV infection [171].

Mucosal delivery of HIV virosomes via intranasal or intravaginal routes has emerged as a promising approach for inducing local IgA and systemic IgG, crucial for blocking transmission at mucosal surfaces. In preclinical studies, co-administration with mucosal adjuvants like cholera toxin B subunit yielded strong polyfunctional T-cell and antibody responses in the genital tract and intestinal mucosa [172].

Virosomal HIV vaccines incorporating conserved internal proteins such as Gag or Pol, often coupled with Ni-chelating lipids and TLR agonists, have been developed to promote robust cytotoxic T lymphocyte (CTL) responses. While these formulations have shown potential for long-term viral control in animal models, concerns remain regarding immune exhaustion and off-target inflammation [173].

DNA-encoded HIV immunogens delivered via virosomes have also been investigated, aiming to combine genetic immunization with virosomal targeting of antigen-presenting cells. In mouse and macaque models, these platforms have triggered both systemic and mucosal immunity with enhanced durability of responses. DNA-loaded virosomes elicit long-lived mucosal and systemic immune responses in preclinical HIV vaccine studies [174].

Collectively, these advances highlight the adaptability and promise of virosomal platforms for HIV vaccine and therapeutic development, particularly when combined with potent adjuvants, innovative delivery methods, and multi-target strategies aimed at durable and broad-spectrum protection.

### 4.8. HPV

#### 4.8.1. VLPs

Human papillomavirus (HPV) VLPs remain the foundation of the most successful prophylactic vaccines against HPV-associated cancers. VLPs composed of the major capsid protein L1 spontaneously self-assemble into non-infectious particles that closely mimic the native virus surface, triggering potent neutralizing antibody responses that prevent initial infection. Licensed VLP-based vaccines such as Gardasil^®^, Gardasil-9^®^, Cervarix^®^, Cecolin^®^ and Walrinvax^®^ demonstrate near-universal seroconversion and high efficacy in preventing HPV infection and related cervical lesions across multiple HPV types, underscoring the immunogenic power of VLP platforms in humans. These vaccines are produced in recombinant expression systems and paired with adjuvants to enhance immune responses [175,176].

Recent efforts in next-generation HPV VLP design focus on expanding cross-protection beyond the high-risk types targeted by current vaccines. A promising strategy employs chimeric L1/L2 VLPs, in which conserved epitopes from the minor L2 capsid protein are integrated into the major L1 scaffold. Preclinical studies indicate that these chimeric constructs induce neutralizing antibodies not only against vaccine-included HPV types but also against additional oncogenic strains, overcoming the limitation of type-restricted immunity [177].

Advances in manufacturing and design are also highlighted by work on high-yield production systems for multivalent HPV VLPs. For example, hyper-expression baculovirus platforms have been used to produce quadrivalent HPV L1 VLPs with enhanced yield and structural fidelity, generating robust neutralizing IgG responses in mice comparable to licensed vaccines. This suggests scalable pathways to enhance global vaccine availability, which remains critical for reducing HPV disease burden worldwide [178].

Beyond prophylaxis, there is growing research on therapeutic HPV VLP and peptide-based vaccine formulations aimed at treating existing HPV infections and associated tumors. Although many therapeutic HPV candidates use platforms other than classical VLPs (e.g., DNA, viral vectors, or peptide vaccines targeting E6/E7 oncoproteins), VLP-derived platforms continue to be examined for their ability to present therapeutic antigens or enhanced epitope arrays that could stimulate cytotoxic T-lymphocyte responses important for tumor clearance [178].

HPV VLPs are increasingly explored as modular scaffolds for presenting heterologous antigens. In one study, HPV L1 VLPs displaying HIV-1 envelope trimers activated antigen-specific B cells more effectively than unconjugated antigens and induced dual antibody responses against both HIV and HPV without adjuvant, highlighting their potential as flexible platforms for combinatorial vaccines against sexually transmitted pathogens [179].

HPV VLP vaccine platforms show outstanding immunogenicity and protection but differ in antigen design and application. Classical L1-based VLPs underpin licensed vaccines such as Gardasil^®^, Gardasil-9^®^, Cervarix^®^, Cecolin^®^, and Walrinvax^®^, inducing high neutralizing antibody titers and near-complete protection against targeted HPV types [175,176]. Chimeric L1/L2 VLPs broaden coverage by incorporating conserved L2 epitopes, eliciting cross-neutralizing antibodies against additional oncogenic strains [177]. High-yield baculovirus systems enhance scalability and antigen fidelity for multivalent formulations [178]. Meanwhile, therapeutic and modular HPV VLPs, targeting E6/E7 oncoproteins or presenting heterologous antigens such as HIV envelope trimers, highlight the platform’s versatility for both cancer immunotherapy and next-generation combined vaccines [178,179]. Overall, L1-based VLPs remain the gold standard for prophylaxis, while emerging chimeric and modular designs advance toward broader, multifunctional protection.

#### 4.8.2. Virosomes

One of the most detailed preclinical demonstrations of virosomal HPV vaccines showed that virosomes encapsulating HPV16 E7 protein, a major oncogenic early protein expressed in HPV-associated tumors, could induce strong CD8^+^ T-cell responses and inhibit tumor outgrowth in murine models. In this work, influenza-derived virosomes loaded with soluble HPV16 E7 elicited robust class I MHC-restricted CTL activity and antigen-specific antibody responses, and protected a majority of immunized mice from tumor challenge, underscoring the potential of virosomes as therapeutic HPV vaccine vectors [180].

Despite the promise shown in these preclinical models, virosome-based HPV vaccines have not yet advanced into recent clinical testing in humans, and most work in the therapeutic HPV vaccine arena continues to focus on peptide, DNA, RNA, viral vector, and other nanoparticle platforms. Ongoing reviews emphasize that therapeutic HPV vaccines, which would aim to eradicate established infections and precancerous lesions, are an active area of research with multiple approaches under evaluation, even though no virosomal candidate has yet reached late-stage clinical evaluation [181,182].

### 4.9. Virosomes for Ebola Vaccines

In Ebola research, VLPs are typically engineered by expressing combinations of Ebola structural proteins, most commonly the matrix protein VP40 and the viral glycoprotein (GP) in heterologous expression systems such as mammalian or insect cells [183]. These proteins can spontaneously assemble into particulate structures that resemble authentic virions, exposing conformational epitopes critical for immune recognition [184].

Although much of the foundational work on Ebola VLPs was conducted earlier (e.g., demonstrating that Ebola VLPs containing GP and VP40 protected nonhuman primates against lethal challenge), interest persists in refining VLP design and application. For instance, a bivalent Ebola VLP vaccine incorporating glycoproteins from Zaire and Sudan ebolavirus species elicited strong humoral and cellular responses in animal models, suggesting potential for broader cross-species protection compared with single-strain constructs.

Recent research is focused on understanding and enhancing VLP production itself. Ebola’s VP40 matrix protein alone can form VLPs when expressed in mammalian cells, and computational modeling studies published in 2024 show that co-expression with the Ebola nucleoprotein (NP) may further enhance VLP assembly and budding efficiency. These insights could help optimise VLP yield and structural authenticity, which are important for immunogenicity and scalable manufacturing [185].

Although preclinical evaluation of Ebola VLP vaccines has provided promising immunogenicity data in small and large animal models, no VLP-based Ebola vaccine has yet advanced to late-stage clinical trials. Most current and near-term Ebola vaccine candidates in clinical development involve recombinant viral vectors (such as rVSV-based or adenoviral platforms); however, systematic reviews of Ebola vaccine research note that VLPs remain an important part of the overall vaccine landscape due to their safety and antigen presentation advantages [184].

Given ongoing challenges, such as the antigenic diversity among Ebola virus species and the need for rapid immunogenicity in outbreak settings, continued innovation in VLP engineering (including potential multivalent designs and improved production strategies) may yield next-generation Ebola vaccines that complement existing licensed regimens [184].

### 4.10. Applications and Case Studies: Comparative Overview

VLPs and virosomes have shown broad applicability across viral, bacterial, and parasitic diseases, offering safer and more versatile alternatives to traditional vaccines. Both platforms are biomimetic systems that emulate native virus structures to stimulate potent immune responses without introducing replicative genetic material. However, their mechanisms of immune activation, production systems, and translational challenges differ significantly, leading to complementary advantages in vaccine design and deployment.

#### 4.10.1. Structural and Functional Characteristics

VLPs are protein-based, self-assembled nanostructures that mimic the morphology of authentic virions. Their highly repetitive and ordered surface antigen display efficiently engages B-cell receptors, resulting in strong humoral and T-cell–mediated immunity [9]. They are also capable of presenting multiple epitopes from different pathogens, making them ideal for multivalent or chimeric vaccine constructs [11].

In contrast, virosomes are lipid-based vesicles derived from reconstituted viral envelopes that incorporate viral glycoproteins (such as influenza HA and NA). Their membrane-fusion capability allows efficient antigen or nucleic acid delivery directly into antigen-presenting cells, inducing both systemic and mucosal immune responses [13]. This property makes virosomes particularly valuable for respiratory and mucosal pathogens such as SARS-CoV-2, influenza, and RSV.

#### 4.10.2. Production Systems and Scalability

Production scalability remains a key differentiator between VLPs and virosomes.

VLPs can be efficiently expressed in a variety of systems, including bacterial (*E. coli*), yeast, insect, mammalian, and plant cells, depending on the complexity of the structural proteins and the need for post-translational modifications [24]. Yeast and insect cells are particularly popular due to high yield, safety, and scalability, while mammalian systems are reserved for complex glycoprotein-based VLPs. For example, insect cell-derived H5N1 VLP vaccines have achieved high immunogenicity and cross-protection across viral clades [186].

Virosome production is more complex, requiring the extraction and reconstitution of viral envelopes with incorporated membrane proteins. This process demands rigorous quality control to ensure lipid bilayer integrity and antigen incorporation stability [24]. Consequently, while virosomes excel in adjuvanticity and targeted delivery, they face higher costs and stability challenges during large-scale manufacturing.

#### 4.10.3. Immunogenicity and Adjuvant Use

VLPs exhibit intrinsic immunogenicity due to their highly repetitive antigenic structure, which effectively cross-links B-cell receptors and activates innate immune pathways through pattern-recognition receptor engagement [9]. However, their adjuvant requirements vary:

- Bacterial and yeast VLPs may contain natural TLR ligands, providing self-adjuvanticity [187].
- Mammalian-derived VLPs, though structurally precise, often require adjuvants such as alum, CpG-ODN, or MPLA to boost dendritic-cell activation [188]

Virosomes are less immunostimulatory unless functional viral glycoproteins remain intact. Therefore, adjuvants such as MPLA or CpG-ODN are frequently added to enhance Th1 responses [24,189,190]. Intranasal virosomes combined with chitosan or QS-21 also promote mucosal IgA and tissue-resident memory formation. Licensed examples such as Inflexal^®^ V and Epaxal^®^ rely on the self-adjuvanting properties of influenza HA and NA [14].

#### 4.10.4. Comparative Efficacy in Preclinical and Clinical Studies

VLP-based vaccines induce durable immune protection across diverse pathogens. Single-dose formulations have conferred protection against influenza and Newcastle disease virus in animal models [191]. Virosomal vaccines, such as Inflexal^®^ V and Epaxal^®^, demonstrate excellent safety, high immunogenicity, and enhanced mucosal protection, particularly when paired with adjuvants or mucosal delivery systems [24].

For respiratory pathogens, VLPs provide stable systemic protection through multivalent antigen design, whereas virosomes excel in mucosal delivery and epithelial uptake, supporting needle-free immunization strategies [24,192].

#### 4.10.5. Limitations and Translational Challenges

Although VLPs and virosomes have demonstrated exceptional immunogenicity and safety in preclinical studies, only a limited number have advanced to licensed vaccine candidates. Several key challenges explain this translational gap.

For VLPs, many constructs fail to progress due to:

- Manufacturing and scalability challenges: Complex multi-protein assembly and the need for precise post-translational modifications hinder consistent large-scale production, especially for enveloped VLPs [12].
- Analytical and quantification limitations: Existing viral particle assays (e.g., hemagglutination or SRID tests) lack sensitivity for VLP characterization, impeding regulatory validation [193].
- Low immunogenicity in humans for complex pathogens: Some VLPs, such as those for HIV or HCV, elicit strong responses in animal models but weak or transient immunity in humans due to antigenic variability and immune evasion [164].
- High production costs: Downstream purification and quality control remain expensive, especially for enveloped or glycoprotein-rich VLPs [10].

For virosomes, the main developmental bottlenecks are:

- Complex lipid reconstitution processes: The two-step procedure involving ultracentrifugation and detergent dialysis must preserve membrane integrity and glycoprotein conformation, which complicates reproducibility and industrial scale-up [75].
- Stability issues during storage: Virosomal membranes are prone to lipid oxidation and protein denaturation, reducing shelf-life compared to protein-based VLPs.
- Adjuvant dependency: Virosomes often require immunostimulatory molecules (e.g., MPLA or CpG) for adequate immune activation, increasing regulatory and formulation complexity [24].
- High cost-to-efficacy ratio: Despite clinical success for Epaxal^®^ and Inflexal^®^, many virosome candidates have not been pursued commercially because they do not offer substantial advantages over existing adjuvanted subunit vaccines [15].

Overall, these challenges illustrate that while VLPs and virosomes are scientifically validated platforms, their regulatory, economic, and process complexities have slowed translation into commercial vaccines. Continued progress in cell-free expression, continuous purification systems, and adjuvant optimization is expected to bridge this gap and accelerate their future deployment [12,75].

## 5. Commercial Vaccines and Clinical Trials

The success of VLP-based vaccines such as Gardasil^®^, Cervarix^®^, and Recombivax HB^®^ has established this platform as a standard in prophylactic vaccination against viral infections like HPV and hepatitis B. More recently, the approval of Mosquirix™ (for malaria) and COVIFENZ^®^ (a COVID-19 vaccine) has expanded their applications into parasitic and pandemic pathogens. Virosome-based vaccines like Epaxal^®^ and Inflexal^®^ V have also demonstrated clinical efficacy and stability.

The following table (Table 3) summarizes key commercial vaccines and current clinical trial candidates that utilize VLP and virosome technologies.

VLPs are under clinical evaluation for delivering tumor antigens (e.g., HER2 in breast cancer) and HIV envelope proteins to enhance immune targeting [194]. Plant-based systems are producing VLP vaccines that have entered human trials for influenza and COVID-19, showing promising safety and immunogenicity profiles [195,196].

A VLP-based vaccine for nicotine addiction (NicQb) has completed phase II trials, showing effectiveness in smoking cessation when sufficient antibody levels are induced [197]. Experimental VLP vaccines targeting norovirus and second-generation malaria antigens are progressing in trials, aiming to improve efficacy and immune memory [198,199]. VLPs produced in *Saccharomyces cerevisiae* and *Pichia pastoris* are entering trials for dengue, Zika, and SARS-CoV-2, offering scalable, cost-effective platforms [200].

These next-generation VLP-based vaccines represent a promising frontier in both prophylactic and therapeutic immunization [201].

## 6. Conclusions and Future Directions

VLPs and virosomes represent innovative, safe, and effective biomimetic delivery systems that mimic the structure and immunogenicity of natural viruses without containing infectious genetic material. Their ability to induce robust immune responses makes them highly promising agents for modern vaccinology and immunotherapy.

VLPs are particularly effective for systemic viral infections where strong, durable humoral and cellular responses are essential. Non-enveloped VLPs, composed solely of self-assembled viral structural proteins, provide exceptional stability and reproducibility, making them suitable for vaccines against pathogens such as HBV and HPV, both of which are addressed by licensed VLP-based formulations. Enveloped VLPs, by contrast, incorporate a lipid bilayer derived from the host cell membrane along with viral glycoproteins, allowing them to better mimic native virions and induce both humoral and cellular immunity; these features have been exploited for influenza, RSV, and coronavirus vaccine development in scalable insect or yeast systems. Virosomes, while also possessing a phospholipid bilayer and fusion-active viral glycoproteins, differ fundamentally in that they are reconstituted vesicles lacking internal structural proteins or viral genomes. Their primary role is to act as delivery vehicles: the fusion capability of embedded glycoproteins facilitates targeted antigen or nucleic acid delivery into antigen-presenting cells, enhancing mucosal and systemic responses. This makes virosomes particularly advantageous for mucosal and respiratory pathogens such as influenza, RSV, and SARS-CoV-2.

The optimal choice between a VLP or virosome platform depends on pathogen biology, immune response requirements, and manufacturing context:

- For stable, non-enveloped viruses, VLPs produced in yeast or insect cells provide high yields, robust antigen presentation, and long shelf-life.
- For enveloped or mucosally transmitted viruses, virosomes derived from influenza or other lipid-based templates enable superior mucosal immunity and antigen flexibility.
- Bacterial expression systems are advantageous for simple, non-glycosylated capsid proteins but limited for complex viral antigens.
- Yeast and insect cells balance scalability and correct folding, while mammalian and plant systems are reserved for glycoprotein-rich or complex antigens where authenticity is crucial.

The main limitations of both systems remain in process scalability, downstream purification, and cost-of-goods (COGs). However, recent advances in continuous bioprocessing, glycoengineering, and hybrid VLP–virosome technologies are closing these gaps. Future innovation should prioritize: (i) integrating automated, cell-free expression systems to enhance yield and flexibility; (ii) developing thermostable formulations suitable for low-resource settings; (iii) expanding the use of hybrid and chimeric systems that merge VLP structural precision with virosome delivery versatility; (iv) establishing standardized analytical and regulatory frameworks for nanoparticle vaccines.

Future developments are focused on improving scalability, thermostability, and antigen release control, particularly for deployment in low-resource settings. Hybrid systems and novel adjuvants are being explored to expand functionality and overcome current limitations.

## Figures and Tables

**Table 1:** Comparison of virosomes and VLPs.

Category	Feature	Virosomes	VLPs	Reference
Structural composition	Core composition	Reconstituted viral envelopes composed of phospholipids and viral glycoproteins but devoid of genetic material.	Self-assembled viral structural proteins that form capsid-like or enveloped particles lacking nucleic acids.	[8,12,68]
	Presence of lipid bilayer	Yes; derived from viral envelopes or synthetic phospholipids.	Variable; non-enveloped VLPs lack a lipid bilayer; enveloped VLPs acquire one during budding or assembly.	[14,69,70]
	Genetic material	None (non-infectious).	None (non-infectious).	[14,71]
Mechanistic and immunological features	Mode of delivery	Can fuse with host cell membranes via viral glycoproteins (e.g., HA, F proteins), facilitating antigen or drug delivery.	Enter host cells primarily via receptor-mediated endocytosis; some enveloped VLPs may also fuse with membranes.	[9,68]
	Antigen presentation	Surface glycoproteins are presented in native conformation; lipid bilayer enables encapsulation of additional antigens or adjuvants.	Capsid proteins display repetitive antigenic epitopes in ordered arrays; genetic fusion allows presentation of heterologous epitopes.	[72,73]
	Immune activation	Elicit both humoral and cellular responses by mimicking viral entry and fusion; adjuvants often enhance immunogenicity.	Induce strong B-cell and T-cell activation through multivalent antigen display and innate receptor stimulation; adjuvants used contextually.	[68,74]
Production & scalability	Expression/assembly system	Reconstitution from purified viral components (e.g., HA, NA) with phospholipid vesicles.	Recombinant expression of viral structural proteins in yeast, insect, mammalian, or plant systems, followed by self-assembly.	[12,72]
	Production complexity	Moderate to high; requires membrane reconstitution and detergent removal under controlled conditions.	Variables simpler for non-enveloped VLPs; more complex for enveloped or multi-protein assemblies.	[70,75]
	Time and scalability	Batch-dependent; limited scalability due to ultracentrifugation and detergent dialysis steps.	Scalable; recombinant expression can be optimized for large-scale production (e.g., BEVS, yeast systems).	[12,14]
	Resource and cost considerations	Higher cost due to lipid reagents, purification steps, and stability control.	Generally lower cost; higher yield and shorter production timelines in microbial or insect systems.	[12,14,75]
Overall characteristics	Stability and storage	Moderate stability; sensitive to oxidation and temperature; requires cold chain.	High stability (especially non-enveloped VLPs); suitable for lyophilization and long-term storage.	[72,73]
	Preferred applications	Mucosal vaccines, therapeutic delivery of peptides or drugs.	Prophylactic vaccines for viral infections (e.g., HPV, HBV) and multivalent antigen display platforms.	[8,72]

**Table 2:** NDV VLPs: expression systems, composition, and immunogenicity.

Expression System	VLP Composition/Construct	Animal Model	Immune Response and Protection	Key Features/Advantages	Reference
Insect cells (Sf9)	NDV F protein + Influenza M1 matrix protein	Chickens	100% protection; strong antibody titers; reduced viral shedding	Enabled DIVA (Differentiating Infected from Vaccinated Animals) capability	[137]
Avian cells	NDV NP, M, HN, and F proteins	Mice	Neutralizing antibodies and T-cell responses comparable to inactivated NDV vaccine	Authentic virion-like structure; potential for multivalent antigen incorporation	[138]
Avian cells (Genotype VII strain)	NDV M, HN, and F glycoproteins	Chickens	Complete survival after lethal challenge; IL-2, IFN-γ, TNF-α upregulation	Demonstrated strong cell-mediated immunity	[139]
Plant-based system (*N. benthamiana*)	NDV F and HN proteins		High antibody titers (HI GMT = 6.2 log); cross-neutralizing antibodies against multiple NDV isolates	Rapid, cost-effective, and scalable platform; suitable for developing regions	[140]
Chimeric (Insect or Avian)	NDV F + H5N1 HA (bivalent VLP)		Protective immunity against both NDV and H5N1 influenza	Dual-disease protection; DIVA compatible	[141]

**Table 3:** Commercial vaccines from VLP.

Vaccine Name	Platform	Disease Target	Manufacturer
Gardasil^®^,	VLP	HPV	Merck & Co.
Cervarix^®^	VLP	HPV	GlaxoSmithKline (GSK)
Recombivax HB^®^	VLP	Hepatitis B	Merck & Co.
Engerix-B^®^	VLP		GlaxoSmithKline (GSK)
Sci-B-Vac™	VLP (3rd-gen)	Hepatitis B	VBI Vaccines
Mosquirix™ (RTS,S/AS01)	VLP	Malaria (*Plasmodium falciparum*)	GlaxoSmithKline + PATH
Inflexal^®^ V	Virosome	Influenza	Berna Biotech (Crucell)
Epaxal^®^	Virosome	Hepatitis A	
COVIFENZ^®^	VLP (plant-derived)	COVID-19	Medicago

## Data Availability

No new data were created or analyzed in this study. Data sharing is not applicable to this article.
